# Synthesis: Molecular Structure, Thermal-Calorimetric and Computational Analyses, of Three New Amine Borane Adducts

**DOI:** 10.3390/molecules28031469

**Published:** 2023-02-03

**Authors:** Kevin Turani-I-Belloto, Rodica Chiriac, François Toche, Eddy Petit, Pascal G. Yot, Johan G. Alauzun, Umit B. Demirci

**Affiliations:** 1 Institut Europeen des Membranes, IEM–UMR 5635, ENSCM, CNRS, Universite de Montpellier, 34090 Montpellier, France; 2 Laboratoire des Multimateriaux et Interfaces, UMR CNRS 5615, Université Claude Bernard Lyon 1, 69622 Villeurbanne, France; 3 ICGM, Universite de Montpellier, CNRS, ENSCM, 34293 Montpellier, France

**Keywords:** adduct, amine borane, boranes, boron chemistry, dihydrogen bonds

## Abstract

Cyclopropylamine borane C_3_H_5_NH_2_BH_3_ (C3AB), 2-ethyl-1-hexylamine borane CH_3_(CH_2_)_3_CH(C_2_H_5_)CH_2_NH_2_BH_3_ (C2C6AB) and didodecylamine borane (C_12_H_25_)_2_NHBH_3_ ((C12)2AB) are three new amine borane adducts (ABAs). They are synthesized by reaction of the corresponding amines with a borane complex, the reaction being exothermic as shown by Calvet calorimetry. The successful synthesis of each has been demonstrated by FTIR, Raman and NMR. For instance, the ^11^B NMR spectra show the presence of signals typical of the NBH_3_ environment, thereby implying the formation of B–N bonds. The occurrence of dihydrogen bonds (DHBs) for each of the ABAs has been highlighted by DSC and FTIR, and supported by DFT calculations (via the Mulliken charges for example). When heated, the three ABAs behave differently: C3AB and C2C6AB decompose from 68 to 100 °C whereas (C12)2AB is relatively stable up to 173 °C. That means that these ABAs are not appropriate as hydrogen carriers, but the ‘most’ stable (C12)2AB could open perspectives for the synthesis of advanced materials.

## 1. Introduction

Ammonia borane NH_3_BH_3_ and ethane C_2_H_6_ have one point in common—they are isoelectronic—otherwise they bear important differences, starting from their physical state. Ammonia borane is solid at ambient conditions whereas ethane is gaseous, because of dissimilarities in terms of electronegativity, partial charge of the hydrogen atoms, bond polarity and dipole moment [1]. The intermolecular interactions within the ammonia borane solid are driven by dihydrogen bonds (DHBs) that occur between the acidic hydrogen atoms H^δ+^ of the NH_3_ group and the basic hydrogen atoms H^δ−^ of the BH_3_ group [2]. Such DHBs are stronger than the van der Waals interactions that occur between the almost neutral hydrogen atoms H of CH_3_ of the ethane molecules [3].

Ammonia borane is the ‘lightest’, the fully hydrogenated, representative of amine borane adducts (ABAs), and certainly the best known from the fact that it has been much investigated as a potential hydrogen carrier since the mid-2000s [4]. However, many other ABAs have been developed in parallel, not necessarily for being used as a hydrogen carrier, thus for various uses in chemistry. Examples are follows: ethylenediamine bisborane as hydrogen carrier [5]; diisopropylaminoborane for one-pot borylation [6]; morpholine borane as radical initiator [7]; pyridine borane as reducing agent [8]; ammonia borane as hydrogenation reagent [9]; 1,3,5-(*p*-aminophenyl)benzene-borane as monomer of borazine-linked polymer [10]; and, methylamine borane as precursor of boron-carbon-nitrogen layers [11]. Providing an exhaustive list of the ABAs reported so far and of their potential applications goes beyond the scope of the present article, and for further information, the reader is referred to appropriate review articles [12,13,14,15,16,17,18,19,20].

In the chemistry of the ABAs, DHBs arising between H^δ+^ of NH_x_ (x = 1, 2 or 3) of one molecule and H^δ−^ of BH_3_ of another one play an important role [21]. The examples are as follows. With the aforementioned ammonia borane, the molecules have close intermolecular N−H···H−B contacts owing to DHBs, and resulting in this ABA being solid at ambient conditions [22,23] and that, under moderate heating (ca. 90–110 °C) conditions, H^δ+^ and H^δ−^ react and combine to release molecular hydrogen H_2_ [24,25]. Diammoniate of diborane [(NH_3_)_2_BH_2_]^+^[BH_4_]^−^ is known as the reactive intermediate of ammonia borane [26], and on account of the existence of DHBs, it forms by transfer of H^δ−^ from BH_3_ of one ammonia borane molecule to BH_3_ of another one resulting in the formation of [BH_4_]^−^ and [(NH_3_)_2_BH_2_]^+^, and thus the formation of the ionic [(NH_3_)_2_BH_2_]^+^[BH_4_]^−^ [27]. α-Methylbenzylamine borane C_6_H_5_CH(CH_3_)NH_2_BH_3_ is a chiral ABA that, in chloroform, exists as dimer owing to DHBs [28]. Dodecylamine borane C_12_H_25_NH_2_BH_3_, when solubilized in (anhydrous) tetrahydrofuran, forms stable core-shell aggregates consisting of five self-assembling, dihydrogen-bonded, stretched molecules [29]. Methylamine borane CH_3_NH_2_BH_3_, in the presence of an Ir(III) pincer complex catalyst, dehydrocouples at 20 °C, the process mediated by DHBs and leading to the production of a polyaminoborane of high molecular weight (M_w_ > 20,000) [30]. It is thus clear that, with ABAs, DHBs are ubiquitous. However, Chen et al. [21] noticed that DHBs have not been used to their full potential yet, and they could be further developed in fields such as crystal engineering, self-assembly and synthesis of advanced materials.

**Figure 1:**
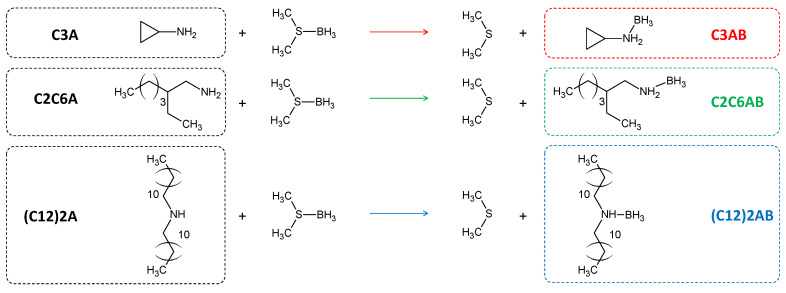
Reaction path for the synthesis of the ABAs in diethyl ether, under argon atmosphere and at ambient temperature: C3AB for cyclopropylamine borane C2C6AB for 2-ethyl-1-hexylamine borane, and (C12)2AB for didodecylamine borane.

Our current research belongs within the context described above. In a first stage, we have been synthesizing and acutely characterizing new ABAs. Recently, we released the results concerning eight alkylamine borane adducts C_n_H_2n+1_NH_2_BH_3_ (*n* = 4, 6, 8, 10, 12, 14, 16 or 18) [31,32]. All have shown higher melting points (by 20–40 °C) than the parent amine C_n_H_2n+1_NH_2_, and such a gain of stability has been explained by the existence of DHBs. For the present study, we considered amines that are not based on monoalkylamines; consequently, we focused on three new ABAs such as cyclopropylamine borane C_3_H_5_NH_2_BH_3_ (C3AB) as a low molecular weight ABA and where the carbonaceous group is cyclic; 2-ethyl-1-hexylamine borane CH_3_(CH_2_)_3_CH(C_2_H_5_)CH_2_NH_2_BH_3_ (C2C6AB) where the carbonaceous chain is an isomer of the n-octyl chain of the C_8_H_17_NH_2_BH_3_ cited above, and didodecylamine borane (C_12_H_25_)_2_NHBH_3_ ((C12)2AB) as the secondary amine analog of the aforementioned C_12_H_25_NH_2_BH_3_. These three new ABAs are synthesized by Lewis acid-base reaction using a commercial amine and a commercial borane complex (Figure 1). They were intensively analyzed by Calvet calorimetry, differential scanning calorimetry, thermogravimetric analysis, Fourier-transform infrared spectroscopy, Raman spectroscopy, ^1^H and ^11^B nuclear magnetic resonance spectroscopy and solid-state ^11^B magic angle spinning magnetic resonance spectroscopy. Their molecular structures were also studied by density functional theory calculations. We had three objectives in mind: to show the occurrence of DHBs; to highlight the properties of ABAs; and to layout the beginnings for their prospect use.

## 2. Results and Discussion

### 2.1. Syntheses of the ABAs Driven by Calvet Calorimetry

**Figure 2:**
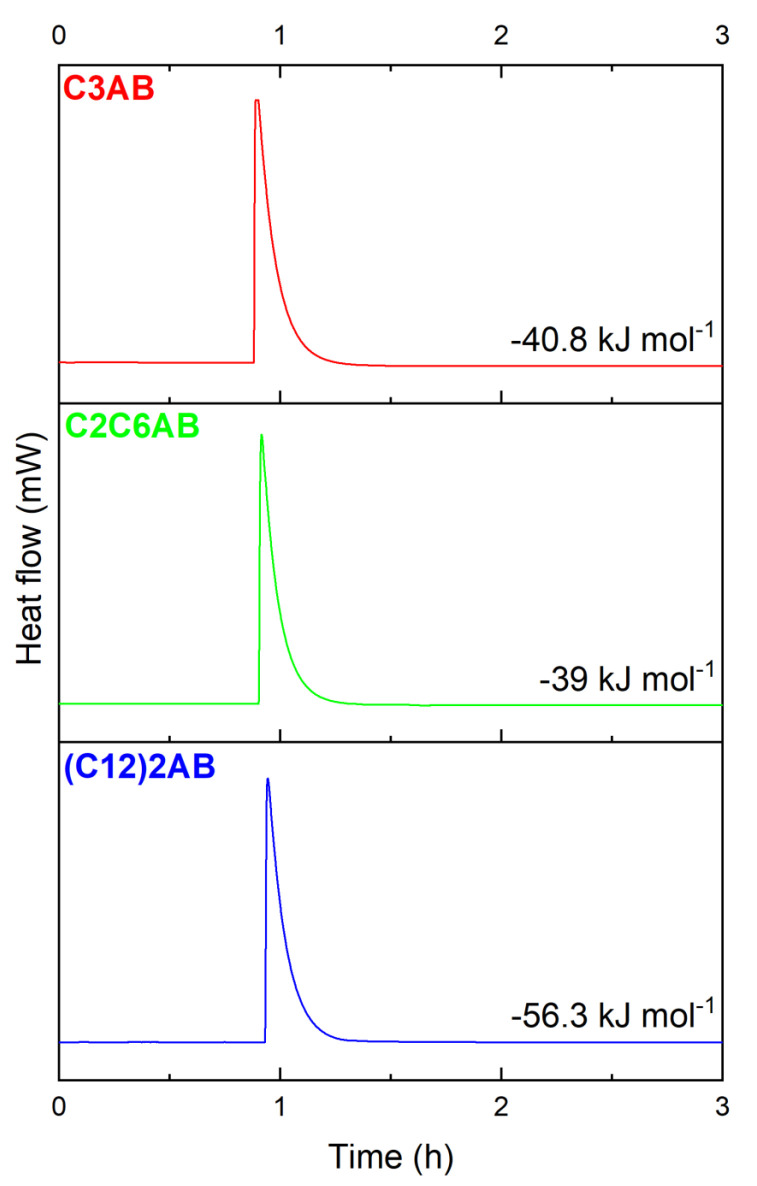
Determination of the enthalpy of reaction by Calvet calorimetry for C3AB, C2C6AB and (C12)2AB.

The amines C3A, C2C6A or (C12)2A readily react with (CH_3_)_2_S·BH_3_. We determined the enthalpies of reaction by Calvet calorimetry (Figure 2). They are of −40.8, −39 and −56.3 kJ mol^−1^ for the syntheses of C3AB, C2C6AB and (C12)2AB, respectively. These enthalpies confirm the exothermic nature of the reaction, as observed during the preparation in the glove box.

**Figure 3:**
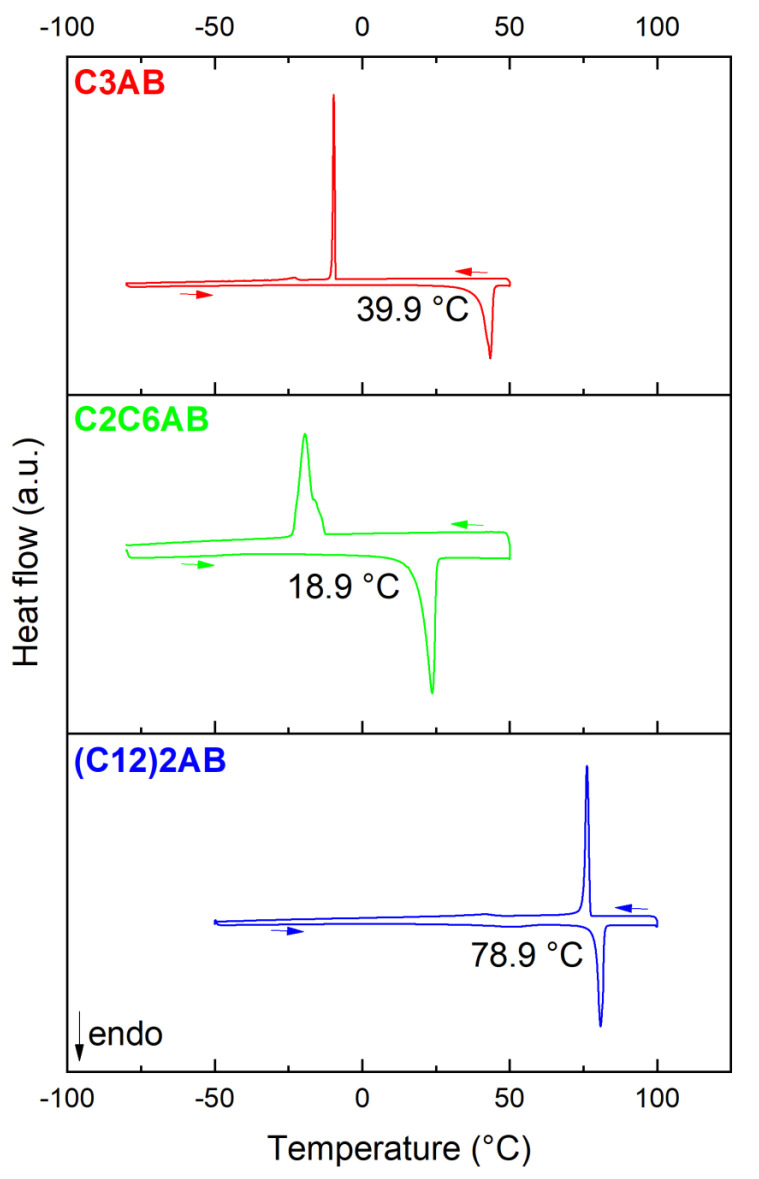
DSC curves of C3AB, C2C6AB and (C12)2AB with the onset temperature of the melting event mentioned.

At room temperature and under argon atmosphere, C3AB is a pasty solid (X-ray diffraction pattern, not reported in this work, is characteristic of an amorphous solid). We determined the onset temperature of the melting event with the help of DSC (Figure 3). When heated from subzero temperatures up to 50–100 °C, an endothermic signal is observed, and it is counterbalanced by an exothermic signal on cooling. This evidences the melting and solidifying of C3AB. The onset temperature of the melting event is 39.9 °C. It is much higher (ΔT of ca. 85 °C) than the melting point of the amine reactant (−45 °C). This indicates additional intermolecular interactions in the ABA, that is, DHBs [21].

C2C6AB is an oily liquid at room temperature. The onset temperature of its melting is 18.9 °C (Figure 3). It is much higher (ΔT of ca. 95 °C) than the melting point of the amine reactant (−76 °C), which is evidence of DHBs occurring between the C2C6AB molecules. It is worth mentioning that, at room temperature, C_8_H_17_NH_2_BH_3_, the n-alkyl analog of C2C6AB, is solid. The melting point of C_8_H_17_NH_2_BH_3_ is higher, with 34.7 °C [29], which can be explained by a lower symmetry (because of the ethyl side group) of the C2C6AB molecule, as is the case with the alkane analogs 2-ethyl-hexane (m.p. −118 °C) and n-octane (−95 °C) [33].

(C12)2AB is a powdery lowly crystalline solid (Figure S1 (Supplementary Materials)). At first glance and with the quality of the pattern, (C12)2AB was found to crystalize into the monoclinic system. The following lattice parameters were determined: *a* = 5.260(1) Å, *b* = 54.232(5) Å, *c* = 4.621(5) Å and β = 109.17(1)°. The space group is possibly the *P*2_1_ (No. 4) one. (C12)2A would then be isostructural with C_8_H_17_NH_2_BH_3_ [31]. The onset temperature of the melting event for (C12)2AB is 78.9 °C (Figure 3), and it is much higher (ΔT of ca. 51 °C) than that of the amine (C12)2A (28 °C). This is due to the occurrence of DHBs between the ABA molecules.

### 2.2. Molecular Characterization of C3AB

**Figure 4:**
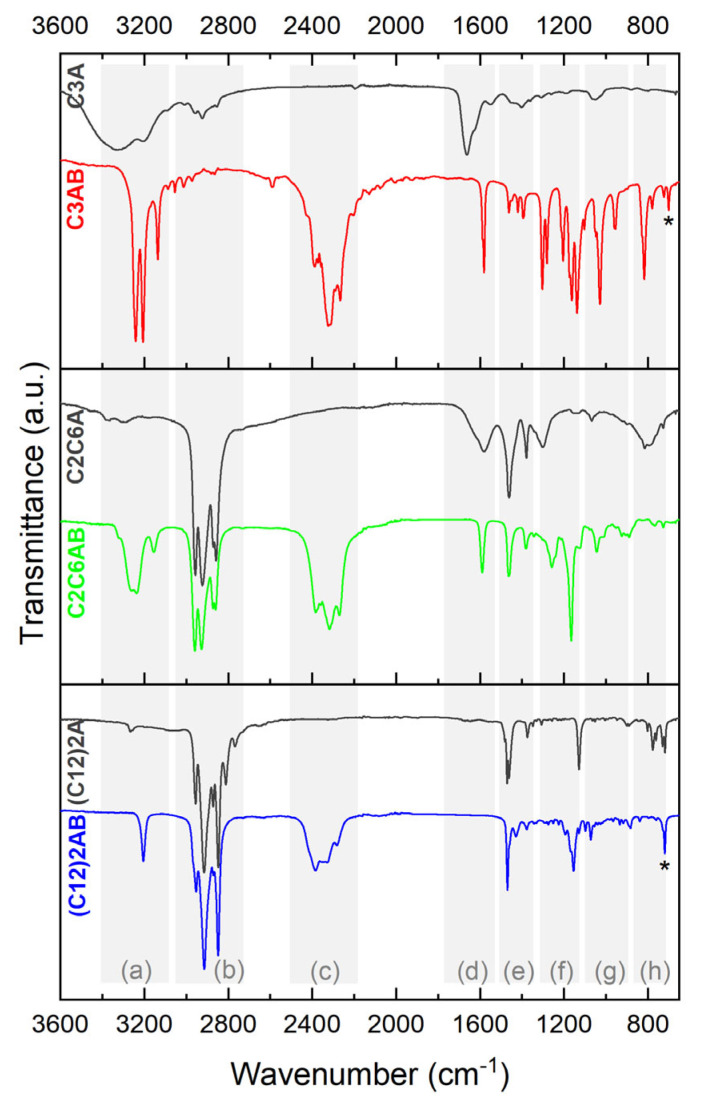
FTIR spectra of C3AB, C2C6AB and (C12)2AB. The spectra of the starting amines C3A, C2C6A and (C12)2A are given for comparison. The bands are attributed to the corresponding vibration modes as follows: (a) N−H stretching; (b) C−H stretching; (c) B−H stretching; (d) N−H deformation; (e) C−H and N−H deformation; (f) B−H deformation; (g) C−N and C−C stretching and C−H deformation; (h) N−H deformation; star for B−N.

C3AB was analyzed by FTIR spectroscopy. The spectrum was compared to that of the amine reactant C3A (Figure 4). There are differences. The spectra were analyzed and the bands were assigned with the help of the references [34,35,36,37]. Bands due to B−H have appeared at 2600–2100 cm^−1^ (stretching) and 1300–1100 cm^−1^ (deformation). The N−H stretching bands (3350–3100 cm^−1^) are more intense and sharper, and they are red-shifted, which is in good agreement with the formation of DHBs between the NH_2_ and BH_3_ groups. Similar changes are observed for the N−H deformation bands (around 1600 and 800 cm^−1^). The bands within the ranges 1500–1300 (C−H deformation for example) and 1100–850 cm^−1^ (C−N and C−C stretching for example) are greater and sharper [37]. The B−N bond is featured by a stretching vibration at about 700 cm^−1^. Otherwise, the spectrum of C3AB favorably compares with the one we predicted by DFT (Figure S2).

**Figure 5:**
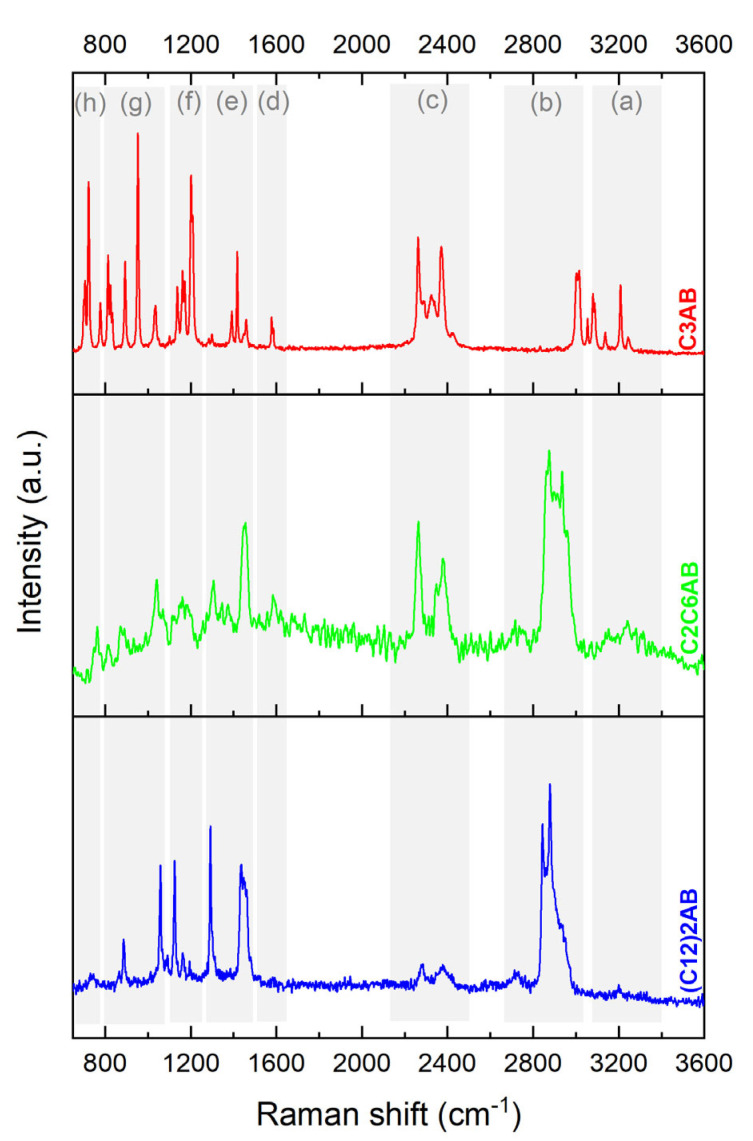
Raman spectra of C3AB, C2C6AB and (C12)2AB. The bands are assigned: (a) N−H stretching; (b) C−H stretching; (c) B−H stretching; (d) N−H deformation; (e) C−H deformation; (f) B−H deformation; (g) C−N and C−C stretching and C−H deformation; (h) B−N stretching.

C3AB was also analyzed by Raman spectroscopy (experimental in Figure 5 and predicted in Figure S3). The spectrum was exploited with the help of the reference [38]. The Raman results (experimental and predicted) are in line with the FTIR obtained, and both allow us to conclude the successful production of C3AB occurred.

C3AB was analyzed by NMR spectroscopy. The ^1^H NMR spectrum (Figure S4) shows four remarkable signals, in good agreement with the molecular structure of C3AB and the chemical shifts predicted by DFT calculations (Figure S5): a quartet of normalized intensity 1:1.1:1.1:1 at 1–1.7 ppm (^1^J_B−H_ of 95.6 Hz) typical of BH_3_; a signal at 4.05 ppm due to the 2 H of NH_2_; a multiplet at 0.61 ppm due to the 4 H of the cyclopropyl’s CH_2_ groups; and, a septuplet at 2.25 ppm due to the H of CHNH_2_. Based on these data, the purity of C3AB was found to be ≥98%.

**Figure 6:**
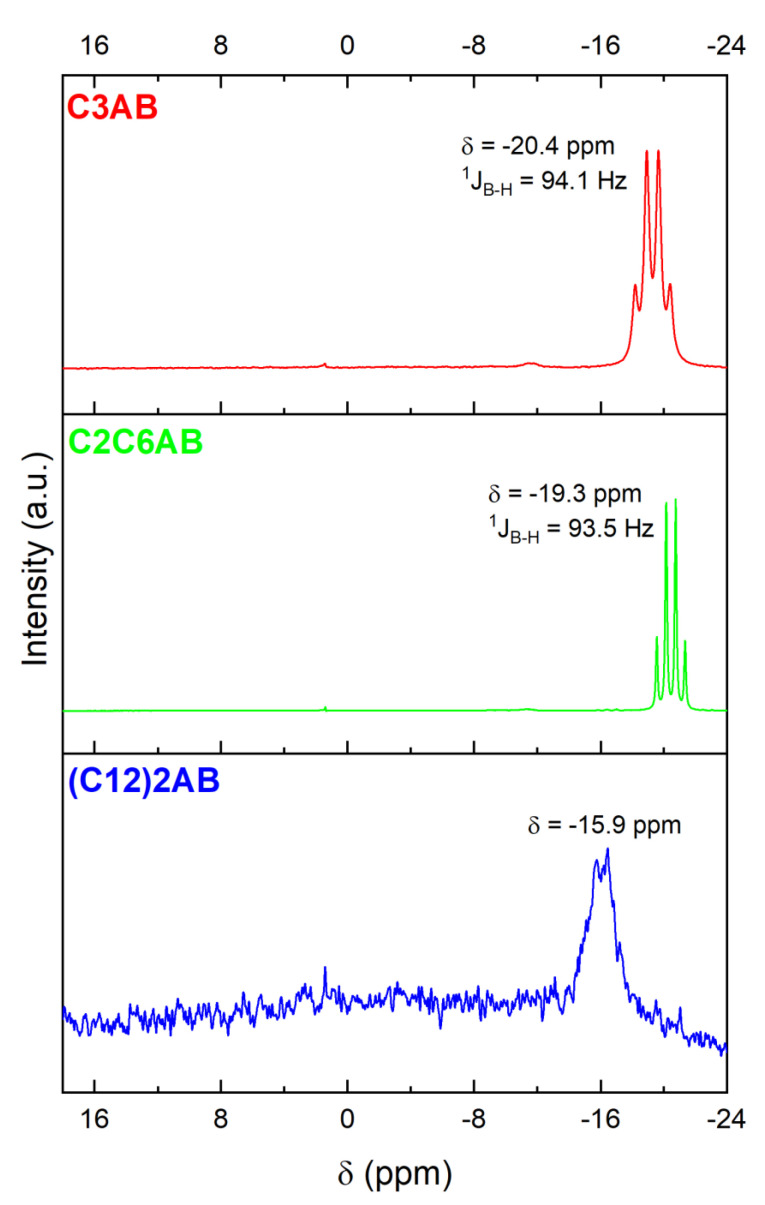
^11^B NMR spectra of C3AB, C2C6AB and (C12)2AB (dissolved in CD_3_CN). The very small signal at 1.4 ppm is due to hydrolysis of the ABAs because of traces of water in CD_3_CN. The very small signal at −11.5 ppm for C3AB is ascribed XBH_3_ (with X = OH^−^from H_2_O, CH_3_CN from CD_3_CN or (CH_3_)_2_S) (i.e., the borane used as reactant).

The ^11^B NMR spectrum (Figure 6) shows a quartet at −20.4 ppm, of normalized intensity 1:2.5:2.5:1, and with a coupling constant ^1^J_B−H_ of 94.1 Hz [39,40]. It is favorably due to the NBH_3_ environment of C3AB.

**Figure 7:**
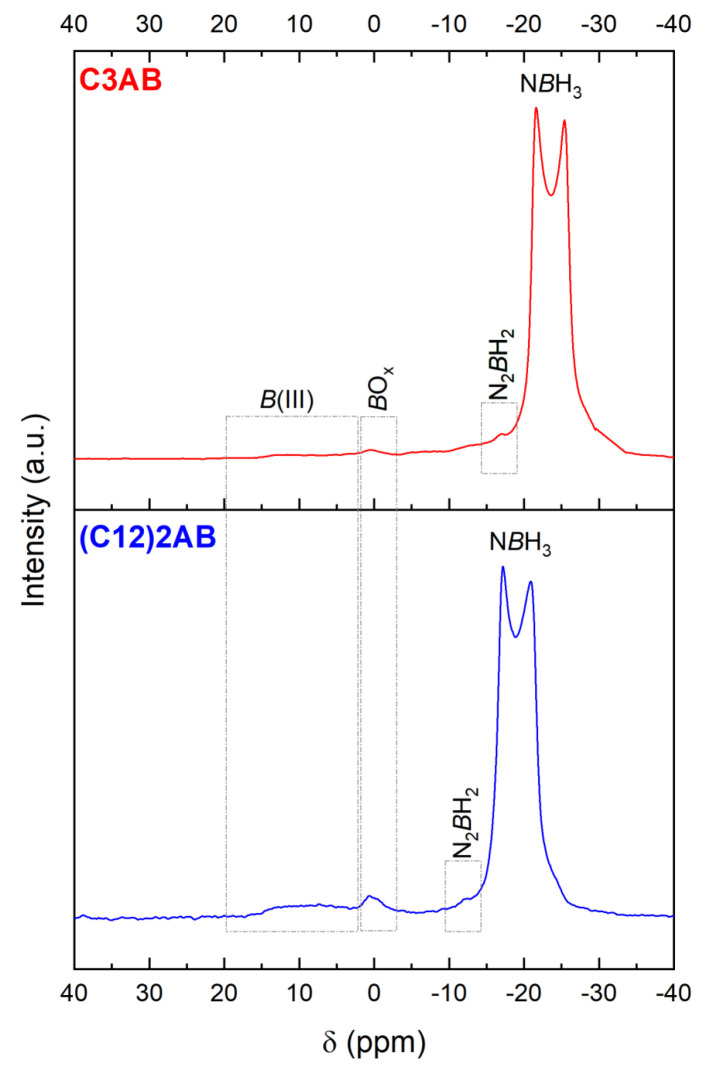
^11^B MAS NMR of the ABAs in solid state, i.e., of C3AB and (C12)2AB. The signals are assigned, and discussed in the main text.

The ^11^B MAS NMR spectrum of C3AB is typical of the spectrum of an ABA (Figure 7), showing a double-horned peak due to NBH_3_ [41]. The signal is centered −23.5 ppm. There are other signals of much smaller intensity (at −16.9, 0.4 and 5–15 ppm). They are attributed to the N_2_BH_2_, BO_x_, and B(III) environments [42,43]. They are likely to be due to dehydrocoupling and hydrolysis of C3AB, but to a very small extent. The former reaction may have taken place during analysis and rotor rotation, and the latter one because of a slight moisture contamination (when transferring the rotor from the lab to the NMR apparatus located in another building) given that ABAs are moisture sensitive [29].

### 2.3. Molecular Characterization of C2C6AB

The FTIR spectrum of C2C6AB (Figure 4) shows [34,35,36,37]: (i) the vibration modes of the B−H bonds (stretching between 2600 and 2100 cm^−1^ and deformation at 1200–1100 cm^−1^); (ii) red shifted and more intense bands for the N−H stretching (3400–3100 cm^−1^); (iii) less intense bands for the N−H deformation (around 800 cm^−1^); and (iv) the vibration modes of the C−H, C−C and C−N bonds as for the amine C2C6A. The B−N stretching band cannot be properly assigned as overlapping the N−H deformation bands. The spectrum favorably compares to the predicted one (Figure S6).

C2C6AB was also analyzed by Raman spectroscopy (experimental in Figure 5 and predicted in Figure S7) [38]. The results are in line with the FTIR ones. These observations allow us to conclude the successful production of C2C6AB occurred.

C2C6AB was analyzed by NMR spectroscopy. The ^1^H NMR spectrum (Figure S8) shows eight signals in good agreement with the molecular structure of the analyzed ABA and the chemical shifts predicted by DFT calculations (Figure S9): namely, a multiplet at 0.8–1.8 ppm due to the 3 H of BH_3_; a triplet at 0.92 ppm due to the 3 H of CH_3_ of the main alkyl chain that is partly overlapping a triplet at 0.88 ppm due to the 3 H of CH_3_ of the ethyl chain; a singlet of high intensity at 1.28 ppm due to the CH_2_ groups of the hexyl chain between CH_3_ and the beta CH_2_; a multiplet at 1.34 ppm due to the CH_2_ of the ethyl chain; a multiplet at 1.56 ppm due to the H of CHCH_2_N; a quintet at 2.57 ppm due to the 2 H of CH_2_N; a broad singlet of low intensity at 3.83 ppm due to the H of NH_2_. Based on these data, the purity of C2C6AB was found to be ≥99%.

The ^11^B NMR spectrum (Figure 6) shows the quartet due to NBH_3_ of the C2C6AB [39,40]. It is located at −19.3 ppm, is of normalized intensity 1:2.6:2.6:1 and has a coupling constant ^1^J_B−H_ of 93.5 Hz.

### 2.4. Molecular Characterization of (C12)2AB

(C12)2AB was analyzed by FTIR spectroscopy (Figure 4), and its spectrum favorably compares to the predicted spectrum (Figure S10). As for the aforementioned ABAs, there are differences between the spectrum of (C12)2AB and that of (C12)2A. The main features are as follows [34,35,36,37]. The N–H stretching band has red-shifted with the addition of the BH_3_ group by 60 cm^−1^ (3207 cm^−1^ for (C12)2AB and 3267 cm^−1^ for the starting amine). Another remarkable change is for the N–H deformation mode between 800 and 700 cm^−1^: the number of bands has decreased with the addition of BH_3_. This can be explained by the presence of DHBs that impose constraints on the N−H bond [35]. The band labelled by a star belongs to the B−N bond.

Similar observations can be made from the Raman spectrum (experimental in Figure 5 and predicted in Figure S11). These results confirm the successful production of (C12)2AB.

(C12)2AB was analyzed by NMR spectroscopy. The ^1^H NMR spectrum (Figure S12) shows six signals: a multiplet at 0.5–2 ppm due to the 3 H of BH_3_; a triplet at 0.91 ppm due to the 6 H of the two CH_3_; a singlet of high intensity at 1.31 ppm due to the eighteen CH_2_ groups between CH_3_ and the beta CH_2_; a multiplet at 1.56 ppm due to the 4 H of [CH_2_CH_2_]_2_N; a quintet at 2.61 ppm due to the 4 H of [CH_2_]_2_N; a broad singlet of low intensity at 3.89 ppm due to the H of NH. The shifts predicted by DFT calculations (Figure S13) are in line with the experimental data. Based on these data, the purity of (C12)2AB was found to be ≥98%.

The ^11^B NMR spectrum (Figure 6) was systematically recorded with a low resolution because (C12)2AB is lowly soluble in CD_3_CN. Nevertheless, the spectrum shows one main signal which shape suggests a quartet and that is centered at −15.9 ppm. It is due to NBH_3_ of the ABA [44].

The ^11^B MAS NMR spectrum (Figure 7) shows the two-horned peak at −19 ppm featuring NBH_3_ [41]. Like for C3AB, the spectrum shows additional signals of small intensity indicating evolution of (C12)2AB to a small extent (possibly due to dehydrocoupling and hydrolysis because of contamination with moisture).

### 2.5. Further Molecular Analyses by DFT Calculations

The total energy of each of the ABA molecules was determined by DFT calculations. The C3AB molecule has a total energy of −199.97 Hartree. The total energy of the C2C6AB is −397.84 Hartree. It is, in absolute value, higher, which is due to the additional carbon and hydrogen atoms. With respect to the heavier (C12)2AB molecule, the total energy is −1027.04 Hartree.

The DFT calculations allowed extracting the Mulliken charges of the atoms of each of the ABA molecules (Figures S14–S16). The Mulliken charge of the elements B, N, H of BH_3_, H of NH or NH_2_, alpha C and beta C are listed in Table 1. The charges of the hydrogens of the BH_3_ and NH_2_/NH groups are, respectively, negative and positive, thereby confirming the presence of H^δ+^ and H^δ−^ and the occurrence of DHBs between the ABA molecules. The B element is almost neutral for the C3AB and C2C6AB molecules, whereas it has a positive charge in the (C12)2AB molecule. In contrast, the N element has a negative charge in each of the molecules. It is also interesting to mention that the charges of the alpha and beta C elements of the three ABAs are not comparable. For the C3AB molecule, the alpha C element is positively charged and the beta one negatively, but for the C2C6 molecule, it is the opposite and the charges (in absolute value) are bigger. Concerning the (C12)2AB molecule that has two C12 alkyl chains, the two alpha C elements are negatively charged while one of the beta C element has a positive charge and the other one is almost neutral. These differences of charges are also illustrated by the mapped electrostatic potentials (Figures S17–S19).

**Figure 8:**
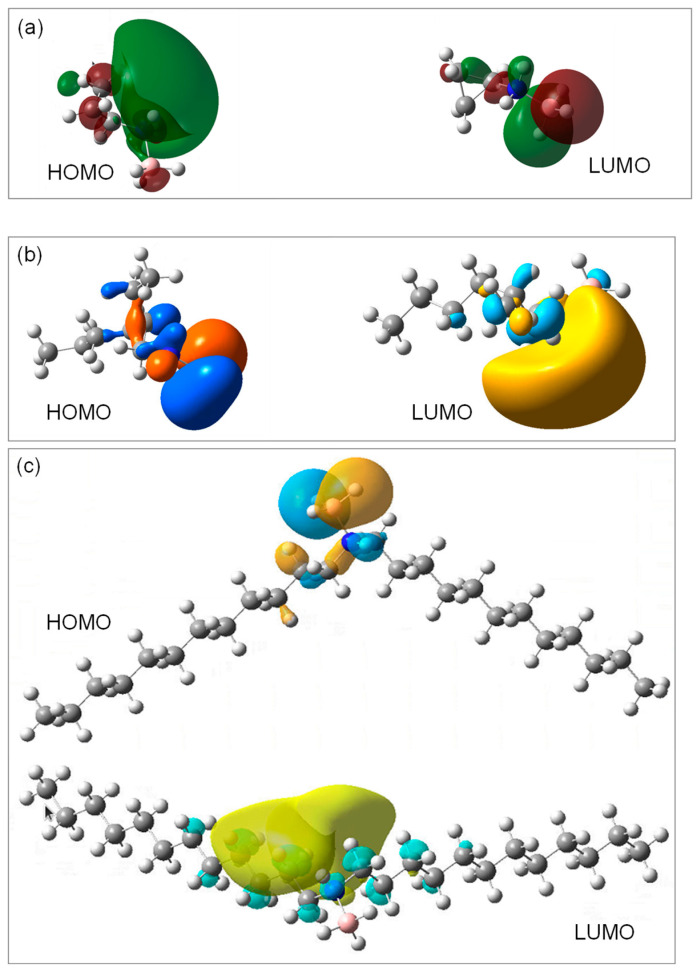
HOMO and LUMO of the (**a**) C3AB, (**b**) C2C6AB and (**c**) (C12)2AB molecules, as predicted by DFT calculations.

The distribution of the highest occupied molecular orbital (HOMO) and that of the lowest unoccupied molecular orbital (LUMO), over the three ABA molecules, were plotted (Figure 8). They are in line with the observations made above about the Mulliken charges. Indeed, the HOMO of the C3AB molecule is mainly localized on the N element as well as on the two beta C elements, and the LUMO is localized on the B element. With respect to the C2C6AB molecule, the HOMO is localized on the hydrogens of the BH_3_ as well as, in a lesser extent, on the C−C−N bonds, and the LUMO is mainly localized on the B−N bond. Finally, the plots obtained for the (C12)2AB indicate a HOMO localized on the BH_3_ group, with a smaller contribution on the N−C bonds, and a LUMO localized onto the C–C–C–C bonds of one alkyl chain bound to N. These observations confirm that the NHBH_3_ and NH_2_BH_3_ groups should be reactive, which is typical of the ABAs [19,45,46], and suggest that the alpha and beta C elements, but not only these carbons in the case of (C12)2AB, are likely to be reactive.

### 2.6. Thermal Properties of the ABAs

Under heating, C3AB decomposes from 68 °C (Figures S20 and S21). Hydrogen is released in two steps over the temperature range 68–200 °C. At about 100 °C, C3AB shows some decomposition with the release and detection of C3A (Figure S22), indicating a breaking of the B–N bond. This could be related to the Mulliken charges of the BN and N elements as well as the localization of the HOMO mainly onto N. Above 150 °C, some NH_3_ is released. The weight loss at 250 °C is of about 85 wt.%, which is higher than the hydrogen content of NH_2_BH_3_ (16.7 wt.%) of C3AB. In other words, pristine C3AB decomposes more than it dehydrogenates.

C2C6AB starts to dehydrogenate from 100.3 °C (Figures S23 and S24). The dehydrogenation is stepwise, with two events peaking at 128 and 179 °C. The weight loss at 200 °C is 8.2 wt.%, which is higher than the 3.5 wt.% of the H of the NH_2_BH_3_ group, thereby suggesting that C2C6AB decomposes more than it dehydrogenates. The main decomposition takes place between 230 and 500 °C. The total weight loss is 97.3 wt.% at 600 °C, because of the release of ammonia together with hydrocarbon fragments (methane and unsaturated C4–C7 species). This decomposition behavior is comparable to that of the previously reported C_8_H_17_NH_2_BH_3_ [31].

(C12)2AB starts to dehydrogenate from about 78 °C but the main decomposition shows an onset temperature of at 173.6 °C (Figures S25 and S26). Up to about 200 °C, the dehydrogenation is featured by two peaks at 154 and 193 °C, and the second dehydrogenation step is concomitant with the decomposition of the alkyl chain. At about 180 °C, (C12)2AB has lost 0.3 wt.%, which corresponds to the release of 0.5 equiv. H_2_. Above 180 °C and up to about 550 °C, (C12)2AB predominantly decomposes though H_2_ is released stepwise (with events peaking at 303, 359, 410 and 474 °C). A number of saturated and unsaturated C4 to C12 fragments (as well as methane), C13 and C14 hydrocarbons due to some fragments combinations and dodecylmethylamine were detected by microGC-MS and GC-MS (Table S1). At 600 °C, the weight loss is 93.3 wt.%, The remaining 6.7 wt.% well matches with the content of B and N (6.7 wt.%) in (C12)2AB, indicating the formation of a boron nitride-based solid.

In the same way as pristine ammonia borane, C3AB and C2C6AB dehydrogenate first, but they then decompose over the temperature range 100–250 °C. Thus, both, in pristine state, cannot be considered as potential hydrogen carriers. They might however have a prospect in this field if combined to an amine, as performed elsewhere [5]. With respect to (C12)2AB, it is more stable. It dehydrogenates from about 100 °C, but the amount of H_2_ released up to 180 °C remains low with 0.5 equiv. H_2_ (over a maximum of 2.5 equiv. H_2_ for the NH_2_BH_3_ group). Such properties are not compatible with those expected for chemical hydrogen storage.

The relative thermal stability of (C12)2AB below 200 °C is interesting when considering synthesis of advanced materials. For instance, polymers using (C12)2AB as monomer could be produced by dehydrocoupling, and such polymers could be used as precursors to produce BCN ceramics by pyrolysis. Another example would be to consider (C12)2AB as a possible soft template to produce nanostructured materials by self-assembly. These two possible applications might also be considered for C3AB and C2C6AB, but one has to keep in mind that they are likely to decompose from 50 to 60 °C.

## 3. Materials and Methods

The reactants, all from Merck, were cyclopropylamine C_3_H_5_NH_2_ (98%; 57.09 g mol^−1^; m.p. −45 °C; denoted C3A), 2-ethyl-1-hexylamine CH_3_(CH_2_)_3_CH(C_2_H_5_)CH_2_NH_2_ (98%; 129.24 g mol^−1^; m.p. −76 °C; denoted C2C6A), didodecylamine (C_12_H_25_)_2_NH (≥97%; 353.67 g mol^−1^; m.p. 28 °C; denoted (C12)2A), and borane dimethyl sulfide (CH_3_)_2_S·BH_3_ (5.0 M in diethyl ether; 75.97 g mol^−1^). The solvent, from Merck also, was anhydrous diethyl ether (≥99.7%). They were all used as received.

The ABAs were synthesized by substitution of (CH_3_)_2_S of the borane complex by one of the amines (Figure 1). Typically, 500 mg of an amine was dissolved in 8 mL of diethyl ether, under stirring (500 rpm) for 1 h, at ambient temperature and under argon atmosphere (glove box MBraun M200B, O_2_ < 0.1 ppm, H_2_O < 0.1 ppm). A slight excess of (CH_3_)_2_S·BH_3_ (1.1 mol versus 1 mol of amine) was added dropwise. Upon a 24-h stirring, the diethyl ether solvent (b.p. 34.6 °C) and the only reaction product (CH_3_)_2_S (b.p. 37.3 °C) were extracted by vacuum distillation at 0 °C in 2 h. In doing so, cyclopropylamine borane C_3_H_5_NH_2_BH_3_ (70.92 g mol^−1^; denoted C3AB), 2-ethyl-1-hexylamine borane CH_3_(CH_2_)_3_CH(C_2_H_5_)CH_2_NH_2_BH_3_ (143.07 g mol^−1^; denoted C2C6AB) and didodecylamine borane (C_12_H_25_)_2_NHBH_3_ (367.5 g mol^−1^; denoted (C12)2AB) were produced.

Calorimetric and thermal analyses were performed using the following techniques. Calvet calorimetry (the C80 model from Setaram) was used to calculate the enthalpy of reaction for the three ABAs from the heat flow monitored against time. The calorimeter uses a reversal stainless-steel hermetic mixing cell with two separated chambers, used for pressures up to 5 bar. In the glove box, the cell was filled so that the 2.5-mL chamber contained the amine solution and the 2-mL chamber contained the borane complex. A reference cell that was kept empty was also used. Both cells were inserted in the calorimeter, and the reaction temperature was fixed at 28 °C. The reactants were mixed by turning the calorimeter, and thus the cells, upside down several times with a rotational motion. The motion was stopped when the reaction peak reached its maximum. The heat flow was monitored against time. It allows for the calculation of the enthalpy of reaction. Differential scanning calorimetry (DSC 1 from Mettler-Toledo) was used for determining the melting points and enthalpies of fusion of the ABA. Indium and mercury were used as standards to calibrate the temperature. Indium and zinc were used to calibrate the enthalpy. This resulted in errors of <1%. Aluminum crucibles (40 µL) were used. They are sealable, which allows preventing the ABAs from air contamination. Typically, the samples to analyze (4 to 7 mg) were prepared in the glove box, and the sealed crucibles were transferred into the DSC oven. The heating rate was 5 °C min^−1^. The nitrogen flow rate was 30 mL min^−1^. A full cycle consisting of a heating step and a cooling step was performed. Thermogravimetric (TG) analysis (TGA/DSC2 from Mettler Toledo) was used for studying the thermal stability and decomposition of the ABA. The analysis conditions were as follows: 15 to 20 mg of ABA; aluminum crucible (100 µL) having a pierced lid; heating of 5 °C min^−1^; and nitrogen flow rate of 30 mL min^−1^. The analyzer was coupled to gas chromatography (GC) and mass spectrometry (MS) detector (7890B GC/5977A MS from Agilent, Les Ulis, France). Two types of couplings can be used: TGA/microGC-MS (SRA Instruments, Marcy l’Etoile, France) to follow hydrogen and other small molecules, and TGA/Storage-Interface/GC-MS for heavier volatile products.

Molecular analyses were performed using the following techniques: Fourier-transform infrared spectroscopy (FTIR; IS50 Thermo Fisher Scientific; from 4000 to 650 cm^−1^; 64 scans; resolution of 4 cm^−1^); Raman spectroscopy (Horiba Jobin Yvon LabRAM 1B; laser Ar/Kr 100 mW 647.1 nm); ^1^H nuclear magnetic resonance spectroscopy (^1^H NMR; Bruker Avance-400 NMR; BBOF probe; CD3CN; 5-mm NMR tube); ^11^B NMR spectroscopy (Bruker AVANCE-400; probe head BBFO; CD3CN; 5-mm tube; 128.378 MHz); and solid-state ^11^B magic angle spinning (MAS) NMR spectroscopy (^11^B MAS NMR; Varian VNMR4000; 128.378 MHz).

For the lowly crystalline (C12)2AB, the lattice parameters were refined by LeBail refinement from diffraction patterns collected at room temperature on a PANalytical X’PERT Pro multipurpose diffractometer (Cu-K_α1_ radiation, λ = 1.54059 Å, 45 kV and 40 mA) equipped with an X’Celerator detector and using Scherrer geometry. The acquisition time was about 10 h. The corresponding powders were loaded into 0.5 mm borosilicate glass capillary tubes in an argon-filled glove box (Jacomex PBOX; O_2_ < 1 ppm, and H_2_O < 2 ppm), and sealed to prevent the samples from moist air contamination.

The molecular structures of C3AB, C2C6AB and (C12)2AB were also studied by density functional theory (DFT) calculations. A gas phase geometry optimization was performed using the DFT/B3LYP method with the 6–311++G (2d, p) basis set available in the Gaussian16 program, which is a good compromise between accuracy and cost. The optimized conformers were calculated at 298.15 K. The FTIR and Raman spectra, as well as the NMR shifts, were simulated and predicted. The Mulliken charges, the electrostatic potentials, the HOMO and the LUMO were calculated.

## 4. Conclusions

The reactions between the selected amines and the borane dimethyl sulfide complex are exothermic (thus spontaneous), as evidenced by Calvet calorimetry. This allowed us to produce three new ABAs, i.e., C3AB, C2C6AB and (C12)2AB, each of them being pure. The formation of a B–N between N of the amine and B of the borane has been confirmed by NMR, FTIR and Raman spectroscopy. For instance, the ^11^B NMR spectra show signals belonging to the NBH_3_ environment that is typical of ABAs.

ABAs are molecules of interest as they are able to interact with each other owing to DHBs. The onset melting temperature of each ABA has been determined by DSC. The values are much higher than the onset temperature of the amine reactants. This is typical of the occurrence of additional intermolecular interactions, that is, of DHBs. By FTIR spectroscopy, a red shift of the N–H stretching bands can be observed when the spectra of the amines and ABAs are compared. This is in good agreement with the existence of DHBs. DFT calculations have given, for each ABA molecule, the Mulliken charges, the electrostatic potentials, the HOMO and the LUMO, and all of these data suggest the occurrence of DHBs between the molecules.

Under heating, C3AB and C2C6AB start to dehydrogenate from 68 to 100 °C and decompose mainly above 100 °C. Unlike these two ABAs, (C12)2AB is more stable, decomposing from 173 °C. With such thermal behaviors, none of these ABAs appears to be appropriate as hydrogen carrier. However, the relative thermal stability of (C12)2AB below 200 °C may open up perspectives for the synthesis of advanced materials.

## Figures and Tables

**Table 1:** Mulliken charges of the elements B, N, H of BH_3_, H of NH_2_ or NH_2_, alpha C and beta C, for the molecules C3AB, C2C6AB and (C12)2AB.

	C3AB	C2C6AB	(C12)2AB
B	−0.028	0.049	0.243
N	−0.449	−0.402	−0.278
H of BH_3_	−0.092 to −0.086	−0.114 to −0.091	−0.131 to −0.109
H of NH_2_ or NH	0.278 and 0.297	0.293 and 0.306	0.278
alpha C	0.156	−0.632	−0.496 and −0.743
beta C	−0.283	0.861	0.298 and −0.017

## Data Availability

Data supporting reported results may be available on demand (to the corresponding author).

## Supplementary Materials

The following supporting information can be downloaded at: https://www.mdpi.com/article/10.3390/molecules28031469/s1, Figure S1. Observed (black line) and calculated (red line) powder XRD profile for the LeBail refinement of (C12)2AB; Figure S2. FTIR spectra of C3AB: predicted versus experimental; Figure S3. Raman spectra of C3AB: predicted versus experimental; Figure S4. ^1^H NMR spectrum of C3AB (in CD_3_CN); Figure S5. Simulated ^1^H NMR chemical shifts for C3AB; Figure S6. FTIR spectra of C2C6AB: predicted versus experimental; Figure S7. Raman spectra of C2C6AB: predicted versus experimental; Figure S8. ^1^H NMR spectrum of C2C6AB (in CD_3_CN); Figure S9. Simulated ^1^H NMR chemical shifts for C2C6AB; Figure S10. FTIR spectra of (C12)2AB: predicted versus experimental; Figure S11. Raman spectra of (C12)2AB: predicted versus experimental; Figure S12. ^1^H NMR spectrum of (C12)2AB (in CD_3_CN); Figure S13. Simulated ^1^H NMR chemical shifts for (C12)2AB; Figure S14. C3AB: partial charges extracted from Gaussian calculations following the Mulliken scheme; Figure S15. C2C6AB: partial charges extracted from Gaussian calculations following the Mulliken scheme; Figure S16. (C12)2AB: partial charges extracted from Gaussian calculations following the Mulliken scheme; Figure S17. Electrostatic potentials of the C3AB molecule; Figure S18. Electrostatic potentials of the C2C6AB molecule; Figure S19. (Electrostatic potentials of the (C12)2AB molecule; Figure S20. TG curve of C3AB superimposed with the evolution of H_2_.as obtained by TGA/microGC-MS; Figure S21. TG (left axis) and DTG (right axis) curves of C3AB; Figure S22. Thermal decomposition of C3AB: evolution of the peak area of the volatile products detected by TGA/microGC-MS coupling as a function of the sampling temperature; Figure S23. TG curve of C2C6AB, superimposed with the evolution of H2 as obtained by TGA/microGC-MS; Figure S24. TG (left axis) and DTG (right axis) curves of C2C6AB; Figure S25. TG curve of (C12)2AB, superimposed with the evolution of H2 as obtained by TGA/microGC-MS; Figure S26. TG (left axis) and DTG (right axis) curves of (C12)2AB; Tableau S1. Volatile organic compounds obtained by coupling TGA with micro GC-MS and GC-MS during the decomposition of (C12)2AB.

## Sample Availability

Samples of the compounds are available from U.B.D., on request for sale on purchase order (after contracting with the CNRS Occitanie Est or the University of Montpellier).
