# Supplementary material for: Synthesis: Molecular Structure, Thermal-Calorimetric and Computational Analyses, of Three New Amine Borane Adducts
Source: Molecules. 2023 Feb 3;28(3):1469. doi: 10.3390/molecules28031469 (PMC9921861; doi:10.3390/molecules28031469)
Supplement: Supplementary file 1 [file molecules-28-01469-s001.zip › molecules-2171765-supplementary.pdf]

Supplementary Materials

Synthesis: Molecular Structure, Thermal-Calorimetric and Computational Analyses, of Three New Amine Borane Adducts

Kevin Turani-I-Belloto [1], Rodica Chiriac [2], François Toche [2], Eddy Petit [1], Pascal G. Yot [3], Johan G. Alauzun [3] and Umit B. Demirci [1,*]

[1] Institut Europeen des Membranes, IEM–UMR 5635, Universite de Montpellier, ENSCM, CNRS,
34090 Montpellier, France; kevin.turaniibelloto@outlook.fr (K.T.-I.-B.); eddy.petit@umontpellier.fr (E.P.)

[2] Laboratoire des Multimateriaux et Interfaces, UMR CNRS 5615, Univ Lyon 1, Université Claude Bernard Lyon 1, F-69622 Villeurbanne, France; rodica.chiriac@univ-lyon1.fr (R.C.);
francois.toche@univ-lyon1.fr (F.T.)

[3] ICGM, Univ Montpellier, CNRS, ENSCM, 34293 Montpellier, France; pascal.yot@umontpellier.fr (P.G.Y.);
johan.alauzun@umontpellier.fr (J.G.A.)

* Correspondence: umit.demirci@umontpellier.fr

Acronyms

C3AB	Cyclopropylamine borane	$C_3H_5NH_2BH_3$
C2C6AB	2-ethyl-1-hexylamine borane	$CH_3(CH_2)_3CH(C_2H_5)CH_2NH_2BH_3$
(C12)2AB	Dodecylamine borane	$[CH_3(CH_2)_{11}]_2NHBH_3$

$(C12)2AB$: $[CH_3(CH_2)_{11}]_2NHBH_3$

Powder X-Ray diffraction results

Despite several attempts (including syntheses at lower temperatures and recrystallization), (C12)2AB was found to be lowly crystalline. The 'best' pattern we collected allowed to determined the lattice parameters and space group from a LeBail fit but was nonetheless exploited to determine the crystal structure (as reported in the article).

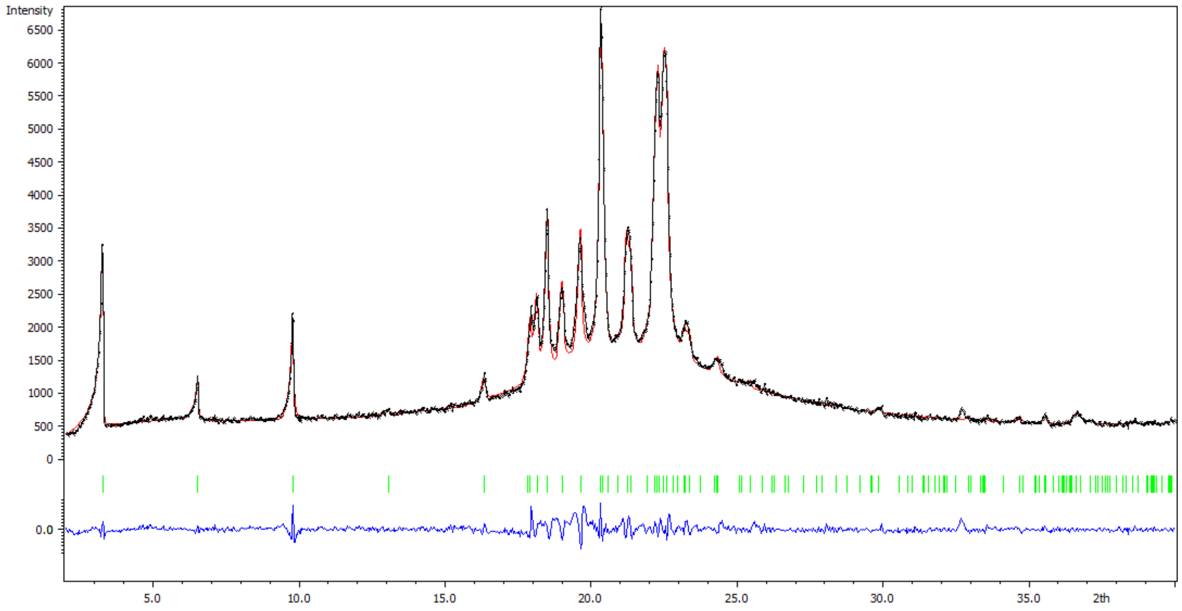

Figure S1. Observed (black line) and calculated (red line) powder XRD profile for the LeBail refinement of (C12)2AB. The bottom curve (in blue) is the difference plot on the same scale intensity and the tick marks (in green) are the calculated angles for the Bragg peaks in 2θ.

C3AB: $C_3H_5NH_2BH_3$

Predicted and experimental FTIR results

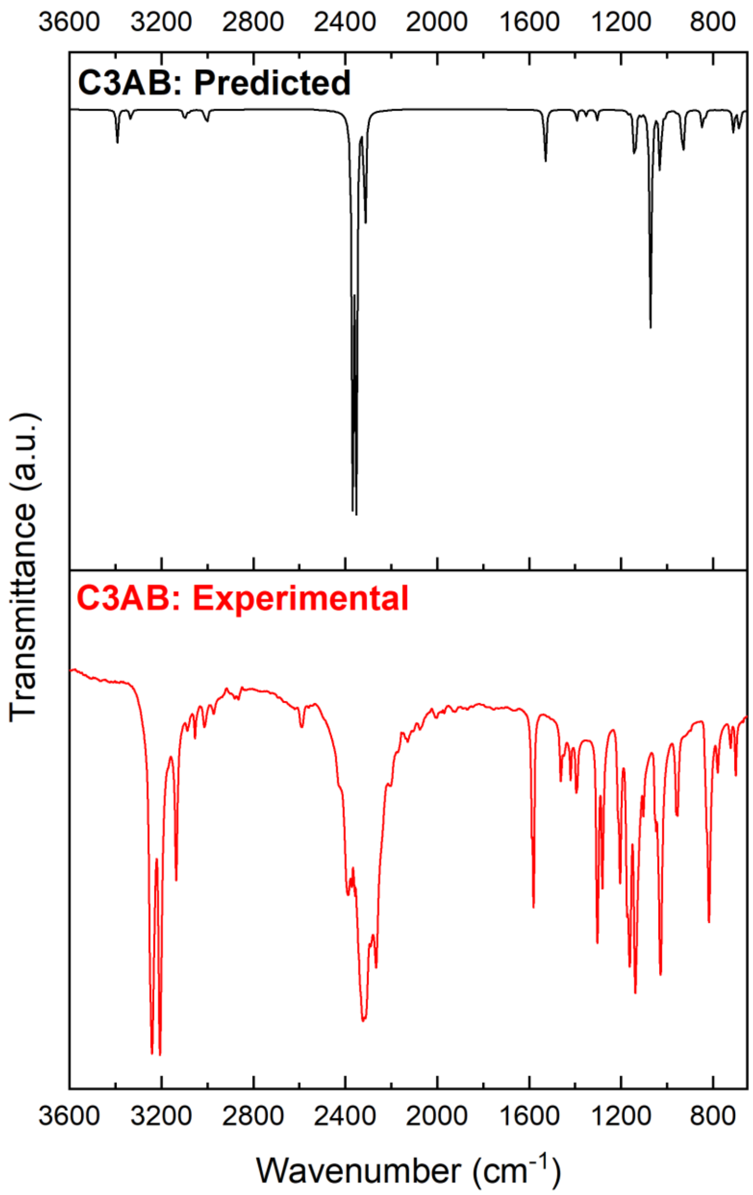

Figure S2. FTIR spectra of C3AB: predicted versus experimental. The predicted vibrational frequencies have not been multiplied by a scale factor (in the range of 0.8 to 1, which may be done to compensate the approximation of the electronic structure calculation and the anharmonicity of the potential energy surface, in order to better match experimental vibrational frequencies).

C3AB: $C_3H_5NH_2BH_3$

Predicted and experimental Raman results

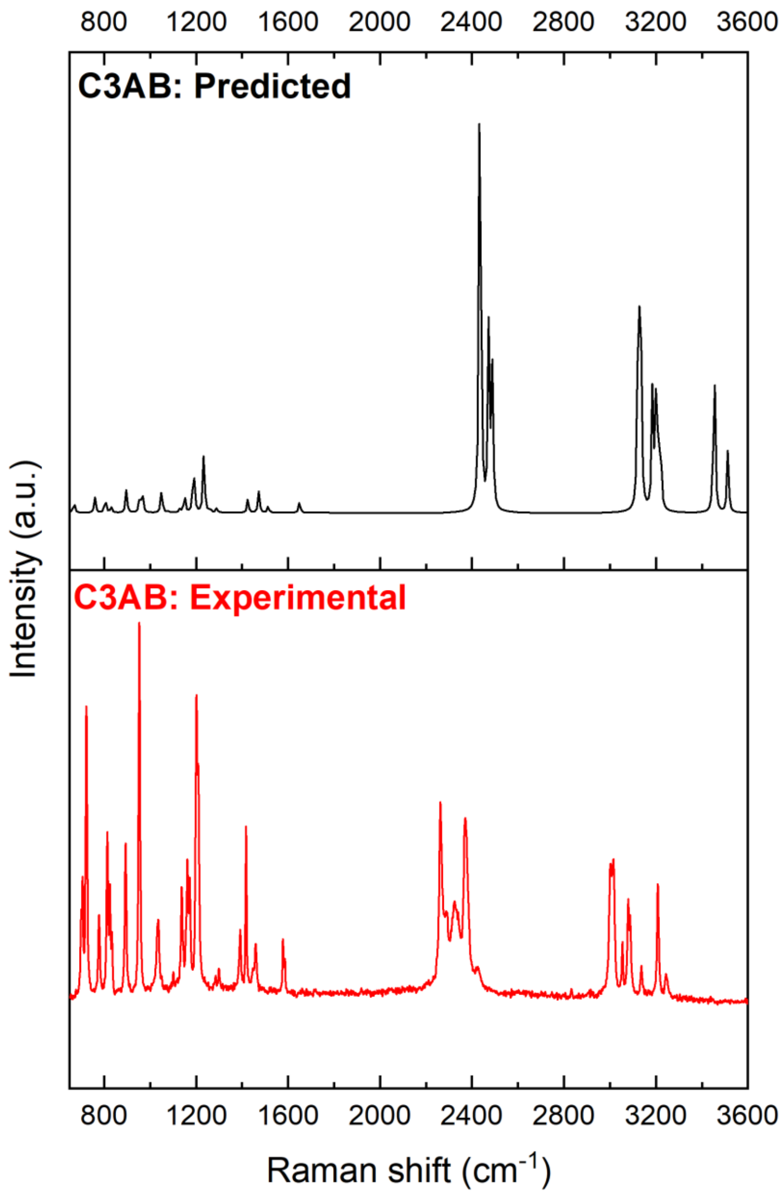

Figure S3. Raman spectra of C3AB: predicted versus experimental. The predicted vibrational frequencies have not been multiplied by a scale factor (in the range of 0.8 to 1, which may be done to compensate the approximation of the electronic structure calculation and the anharmonicity of the potential energy surface, in order to better match experimental vibrational frequencies).

C3AB: $C_3H_5NH_2BH_3$

^{1}H NMR results

$C_3H_5NH_2BH_3$:

- White pasty solid
- Purity, ≥98%
- ^{1}H NMR: δ 1-1.7 (3H, q, 95.6 Hz, B*H*3), 0.61 (4H, m, C*H*2), 2.25 (1H, sept, 6 Hz, C*H*N), 4.05 (2H, m, N*H*2)

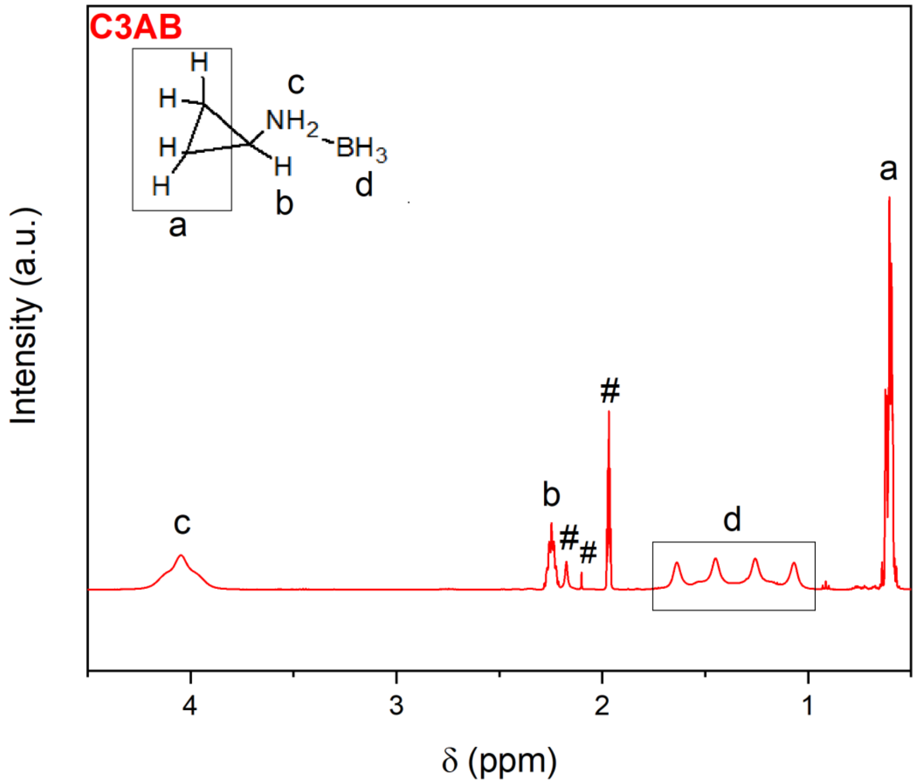

Figure S4. ^{1}H NMR spectrum of C3AB (in CD_3CN). The various signals are assigned. The symbol # indicates the signals due to CH_3CN (1.97 ppm), H_2O (2.15 ppm), and DMS (2.2 ppm).

C3AB: $C_3H_5NH_2BH_3$

Predicted ^{1}H NMR chemical shifts

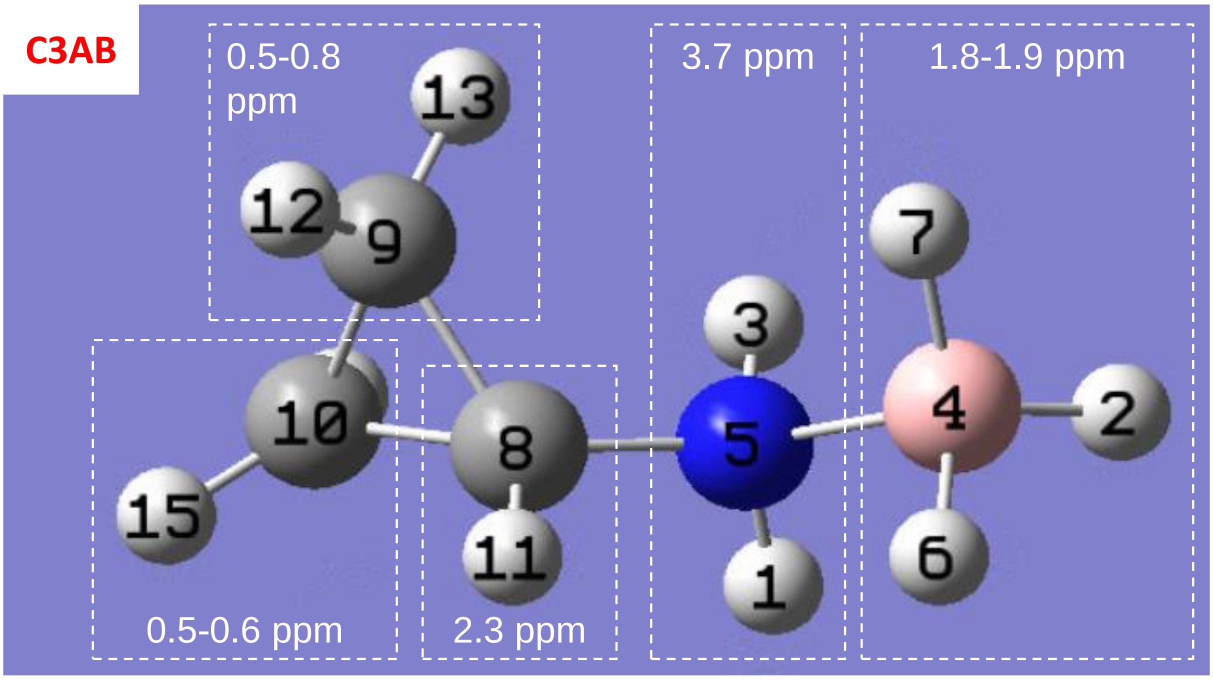

Figure S5. Simulated ^{1}H NMR chemical shifts for C3AB.

C2C6AB: $CH_3(CH_2)_3CH(C_2H_5)CH_2NH_2BH_3$

Predicted and experimental FTIR results

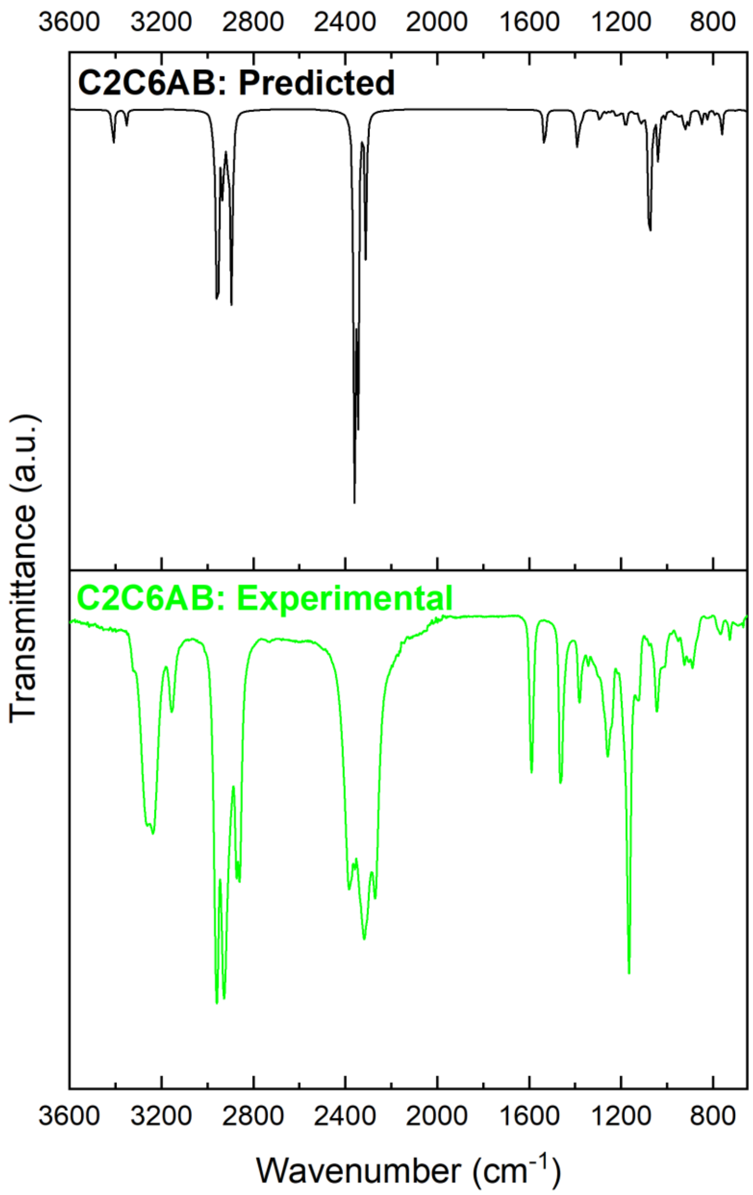

Figure S6. FTIR spectra of C2C6AB: predicted versus experimental. The predicted vibrational frequencies have not been multiplied by a scale factor (in the range of 0.8 to 1, which may be done to compensate the approximation of the electronic structure calculation and the anharmonicity of the potential energy surface, in order to better match experimental vibrational frequencies).

C2C6AB: $CH_3(CH_2)_3CH(C_2H_5)CH_2NH_2BH_3$

Predicted and experimental Raman results

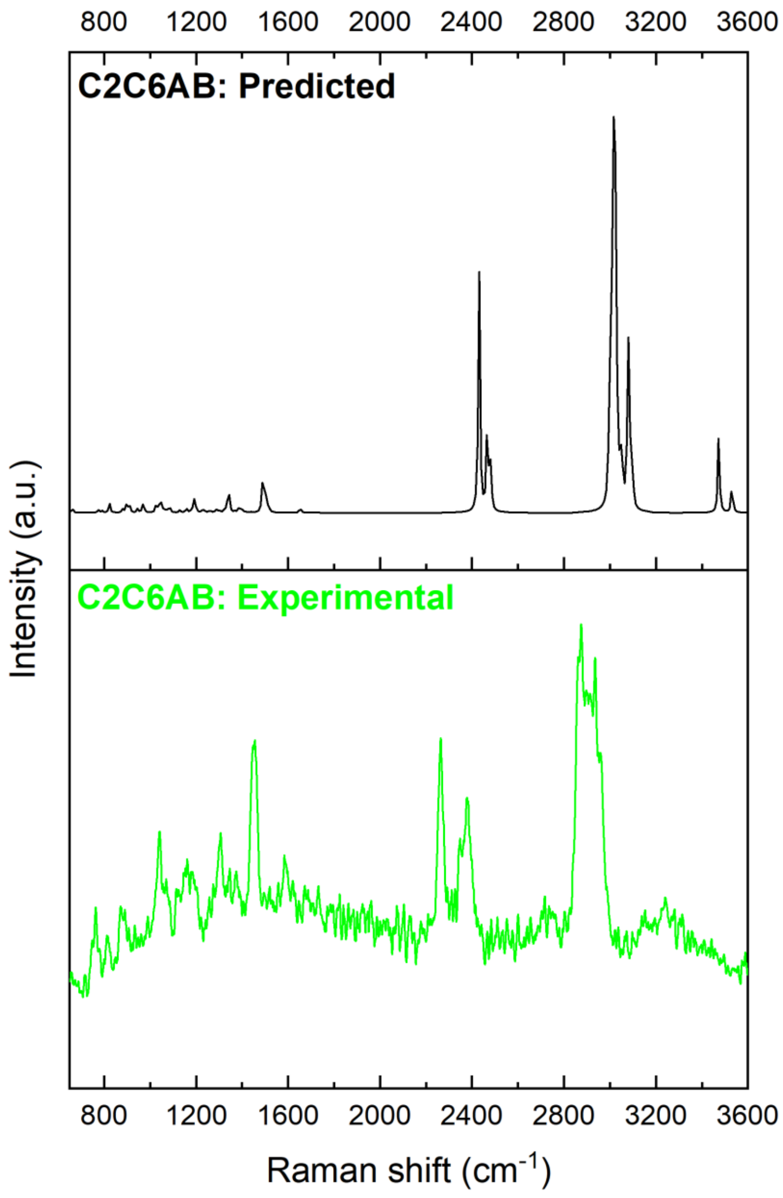

Figure S7. Raman spectra of C2C6AB: predicted versus experimental. The predicted vibrational frequencies have not been multiplied by a scale factor (in the range of 0.8 to 1, which may be done to compensate the approximation of the electronic structure calculation and the anharmonicity of the potential energy surface, in order to better match experimental vibrational frequencies).

$C2C6AB: CH_3(CH_2)_3CH(C_2H_5)CH_2NH_2BH_3$

^{1}H NMR results

$CH_3(CH_2)_3CH(C_2H_5)CH_2NH_2BH_3$:

- Oily liquid
- Purity, ≥99%
- ^{1}H NMR: δ 0.8-1.8 (3H, m, B*H*$_3$), 0.88 (3H, t, 7.4, C*H*$_3$ of C_2H_5), 0.92 (3H, t, 7.1 Hz, C*H*$_3$), 1.28 (6H, s broad, C*H*$_2$), 1.34 (2H, m, CH_2 of C_2H_5), 1.56 (1H, quint, 6.0 Hz, C*H*CH_2N), 2.57 (2H, m, C*H*$_2$N), 3.83 (2H, s broad, N*H*$_2$)

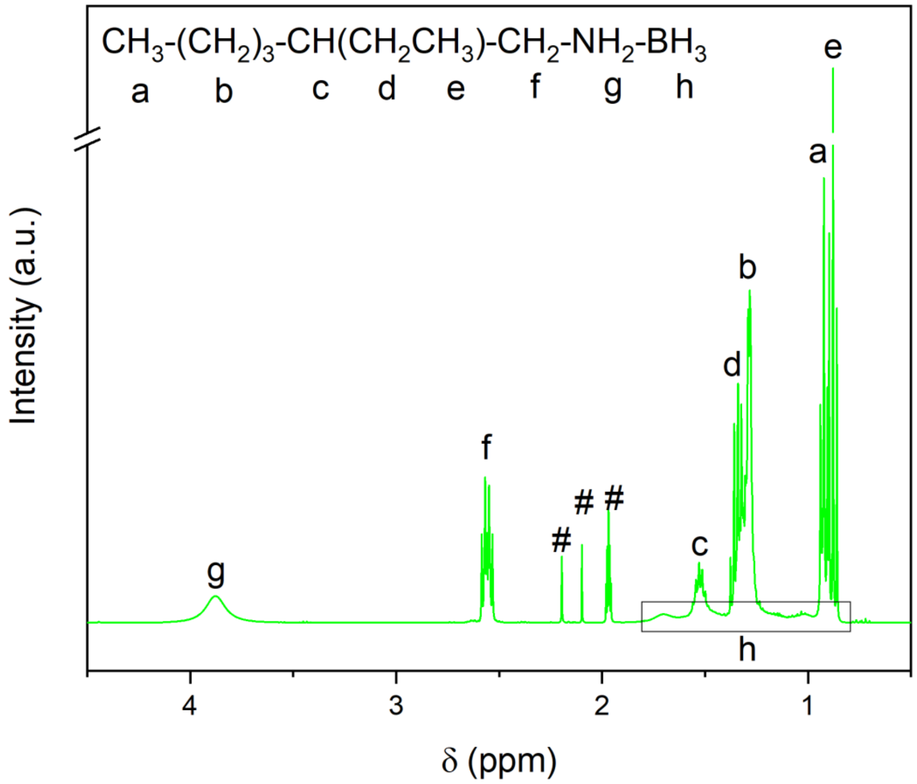

Figure S8. ^{1}H NMR spectrum of C2C6AB (in CD_3CN). The various signals are assigned. The symbol # indicates the signals due to CH_3CN (1.97 ppm), H_2O (2.15 ppm), and DMS (2.2 ppm).

C2C6AB: $CH_3(CH_2)_3CH(C_2H_5)CH_2NH_2BH_3$

Predicted ^{1}H NMR chemical shifts

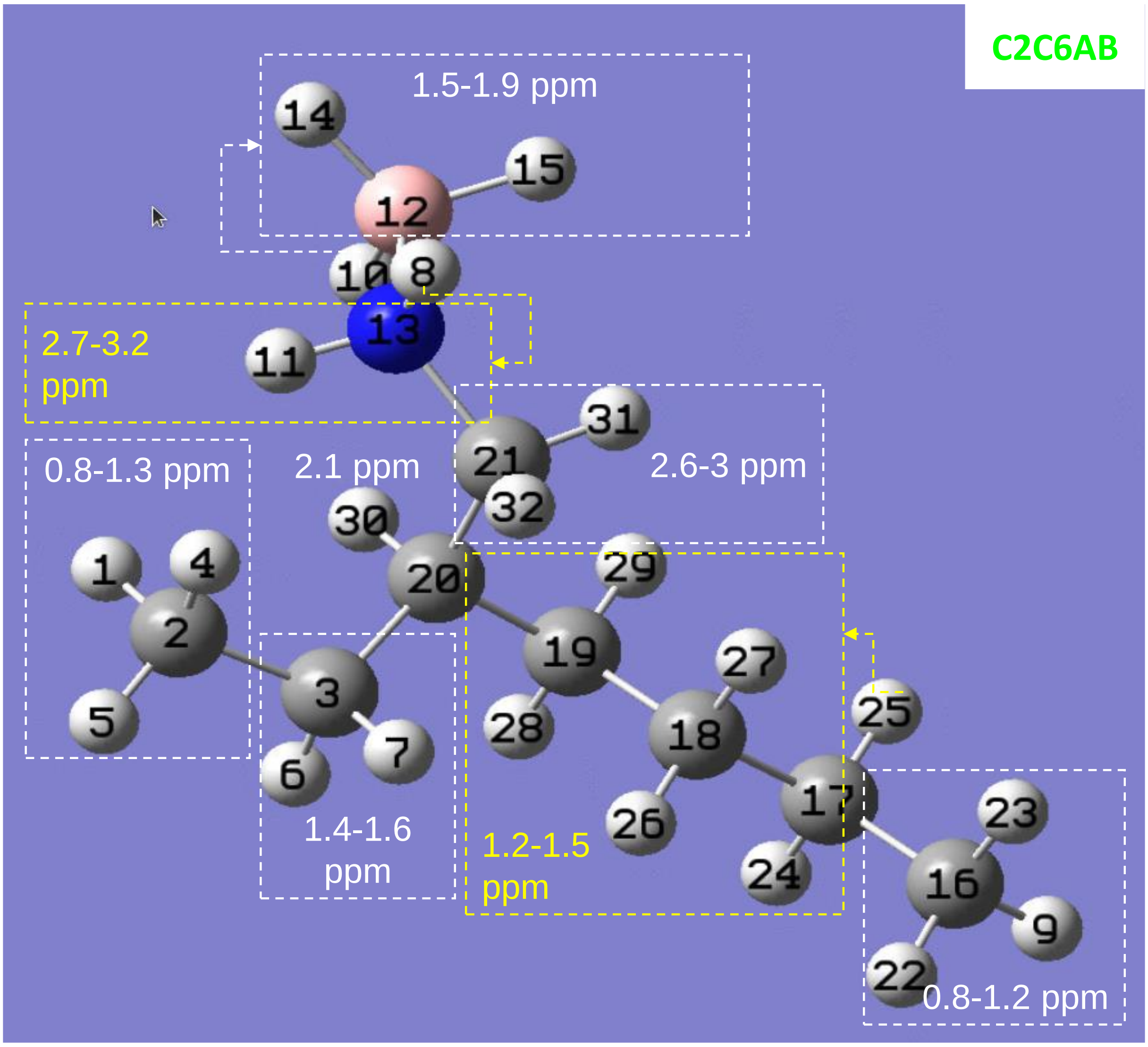

Figure S9. Simulated ^{1}H NMR chemical shifts for C2C6AB.

(C12)2AB: $[CH_3(CH_2)_{11}]_2NHBH_3$

Predicted and experimental FTIR results

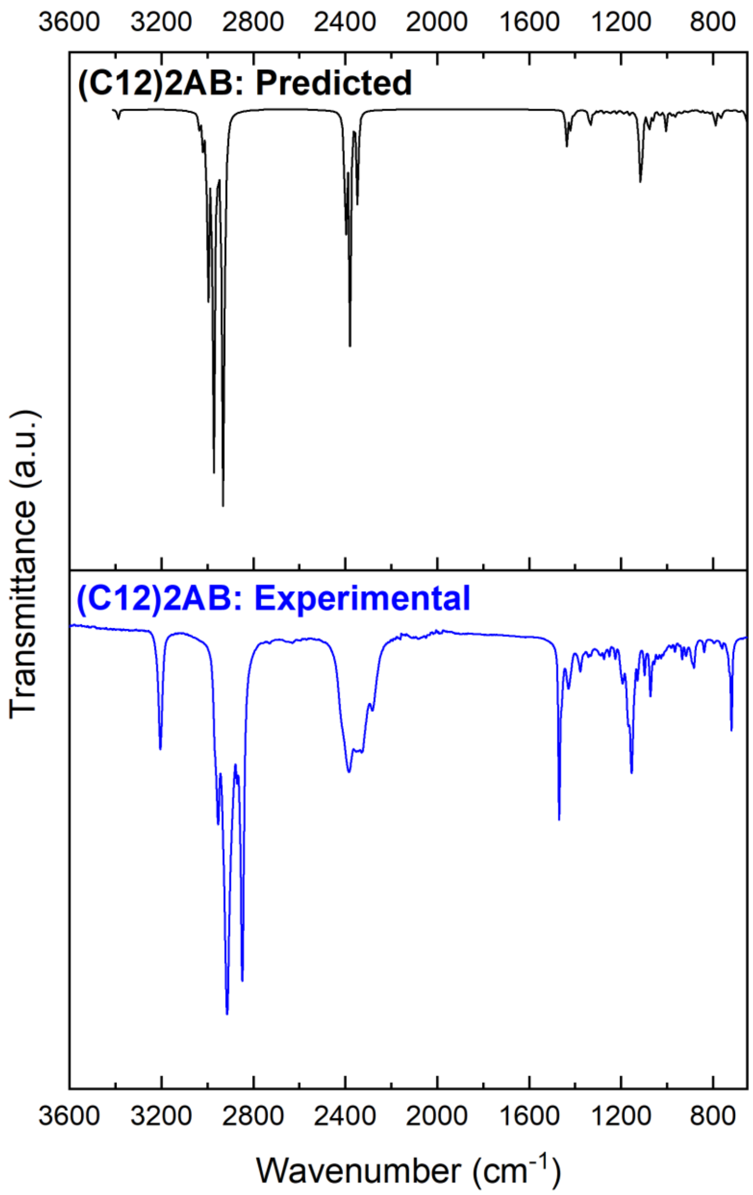

Figure S10. FTIR spectra of (C12)2AB: predicted versus experimental. The predicted vibrational frequencies have not been multiplied by a scale factor (in the range of 0.8 to 1, which may be done to compensate the approximation of the electronic structure calculation and the anharmonicity of the potential energy surface, in order to better match experimental vibrational frequencies).

(C12)2AB: $[CH_3(CH_2)_{11}]_2NHBH_3$

Predicted and experimental Raman results

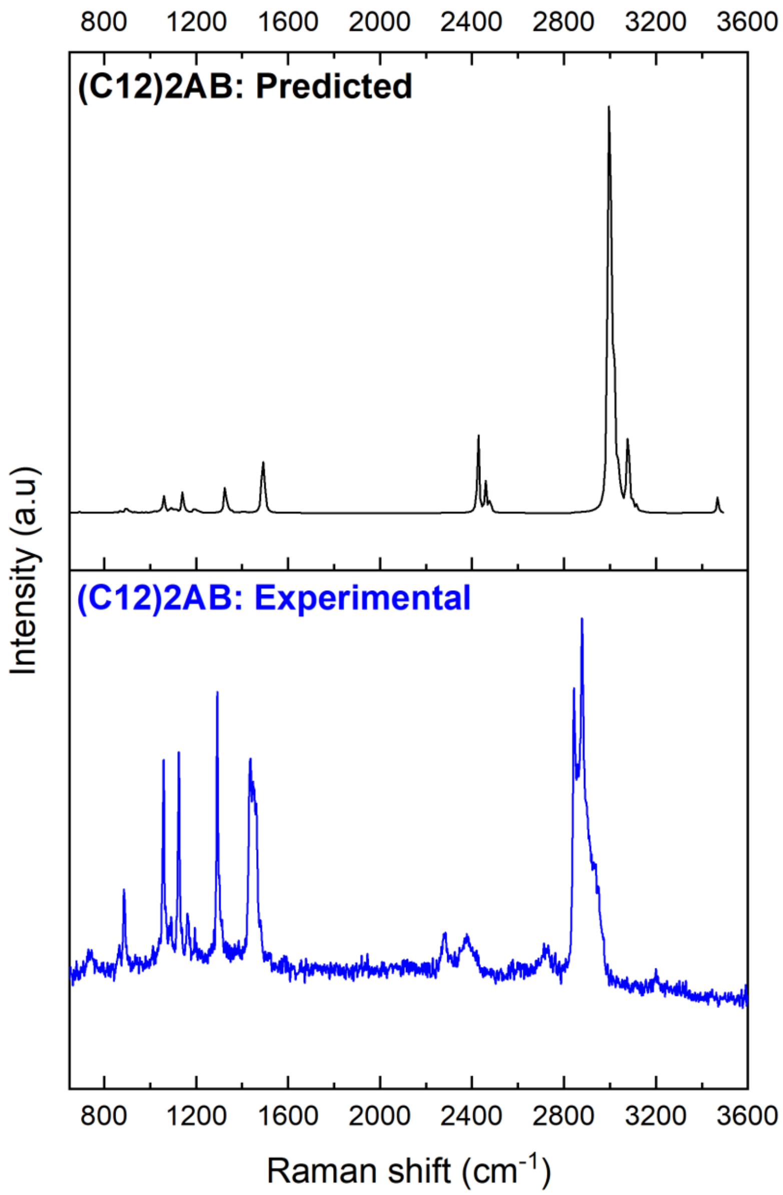

Figure S11. Raman spectra of (C12)2AB: predicted versus experimental. The predicted vibrational frequencies have not been multiplied by a scale factor (in the range of 0.8 to 1, which may be done to compensate the approximation of the electronic structure calculation and the anharmonicity of the potential energy surface, in order to better match experimental vibrational frequencies).

(C12)2AB: $[CH_3(CH_2)_{11}]_2NHBH_3$

^{1}H NMR results

$[CH_3(CH_2)_{11}]_2NHBH_3$:

- White solid
- Purity, ≥98%
- ^{1}H NMR: δ 0.5-2 (3H, m, BH_3), 0.91 (6H, t, 6.7 Hz, CH_3), 1.31 (36H, s broad, CH_2), 1.56 (42H, quint, 6.8 Hz, [CH_2CH$_2$]$_2$N), 2.61 (4H, quint, 7.1 Hz, [CH_2]$_2$N), 3.89 (H, s broad, NH)

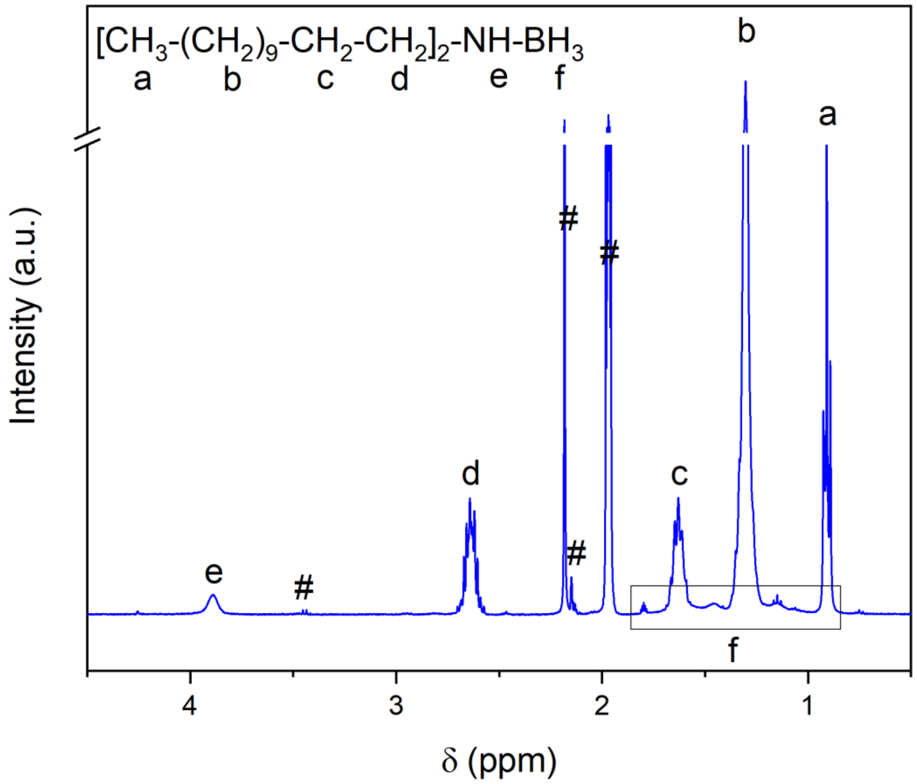

Figure S12. ^{1}H NMR spectrum of (C12)2AB (in CD_3CN). The various signals are assigned. The symbol # indicates the signals due to CH_3CN (1.97 ppm), H_2O (2.15 ppm), and DMS (2.2 ppm).

(C12)2AB: $[CH_3(CH_2)_{11}]_2NHBH_3$

Predicted ^1H NMR chemical shifts

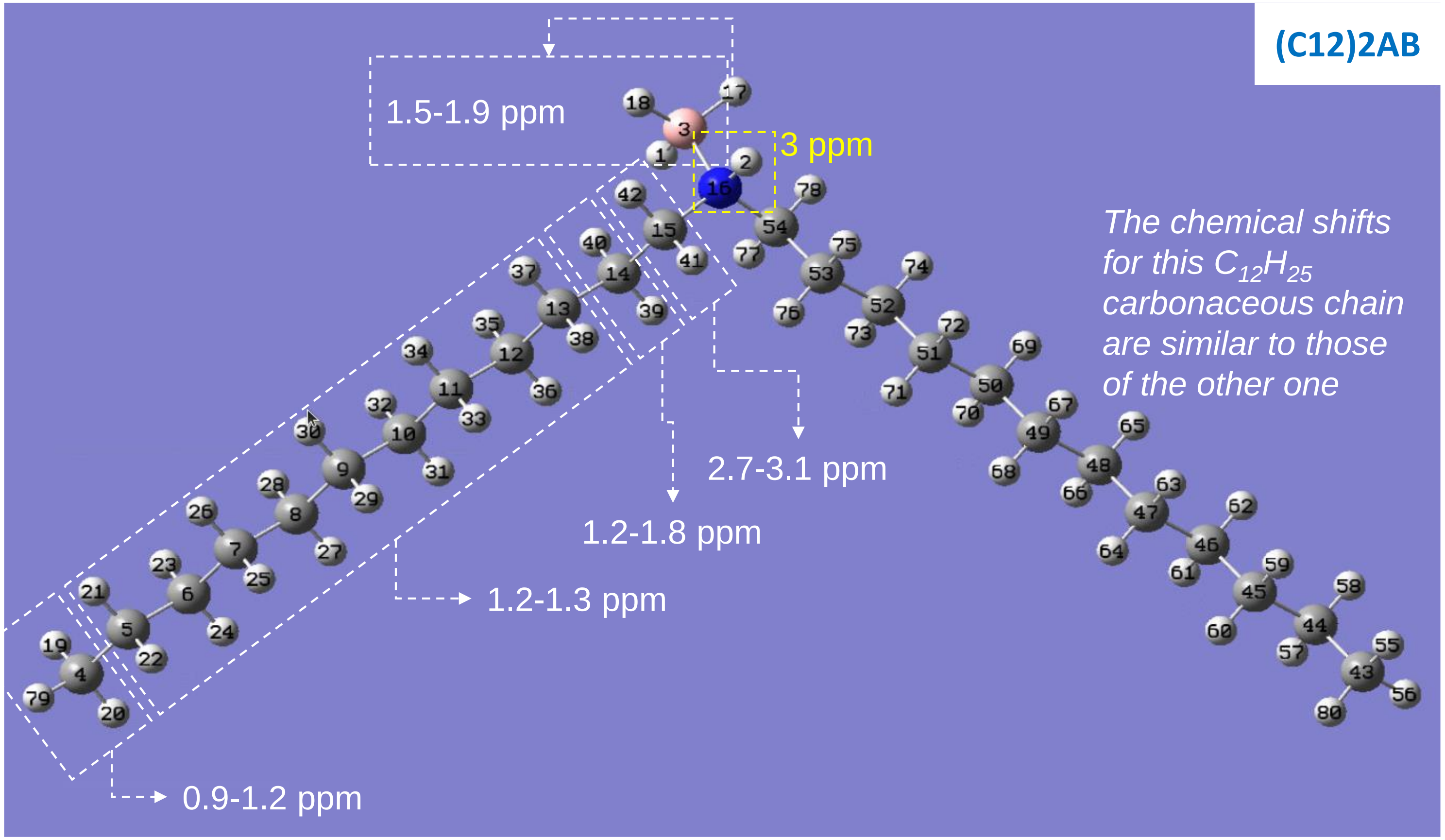

Figure S13. Simulated ^1H NMR chemical shifts for (C12)2AB.

C3AB: $C_3H_5NH_2BH_3$

Mulliken charges from DFT calculations

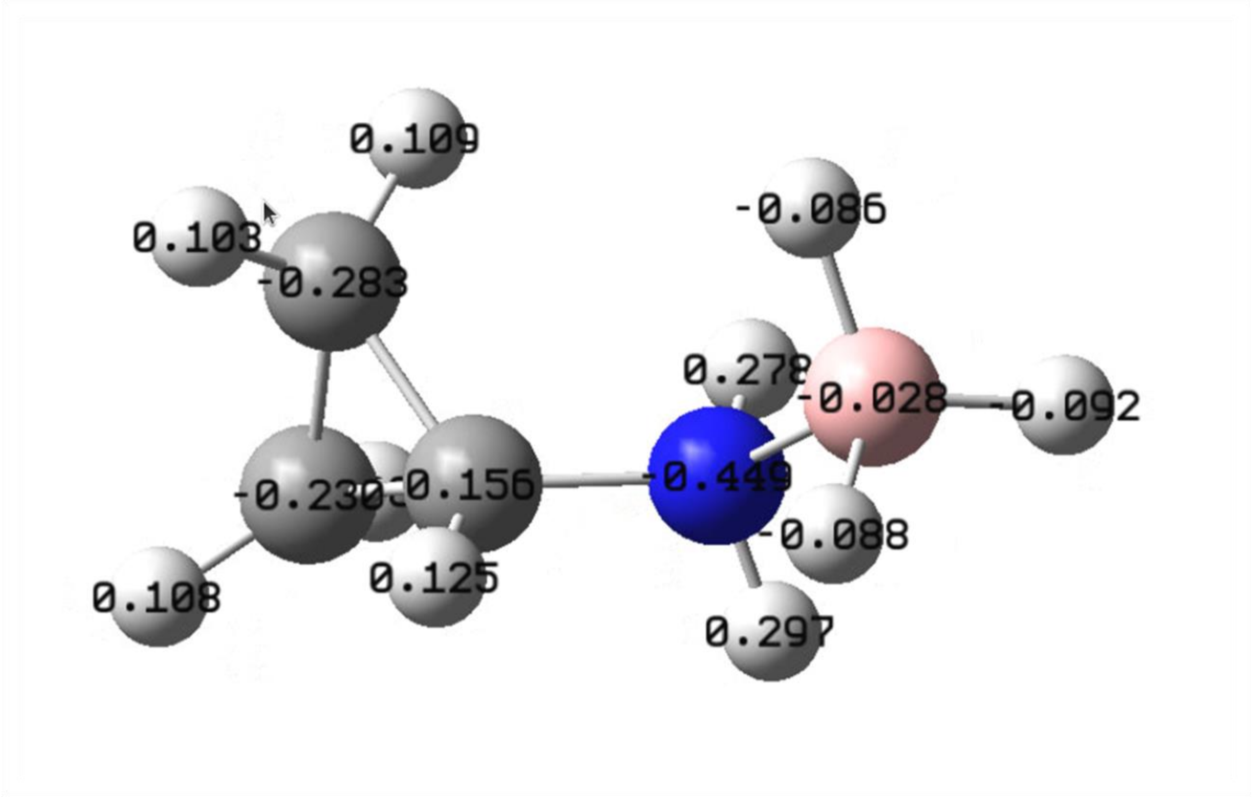

Figure S14. C3AB: partial charges extracted from Gaussian calculations following the Mulliken scheme. The H, B, N and C atoms are represented by white, pink, blue and dark grey spheres, respectively.

C2C6AB: $CH_3(CH_2)_3CH(C_2H_5)CH_2NH_2BH_3$

Mulliken charges from DFT calculations

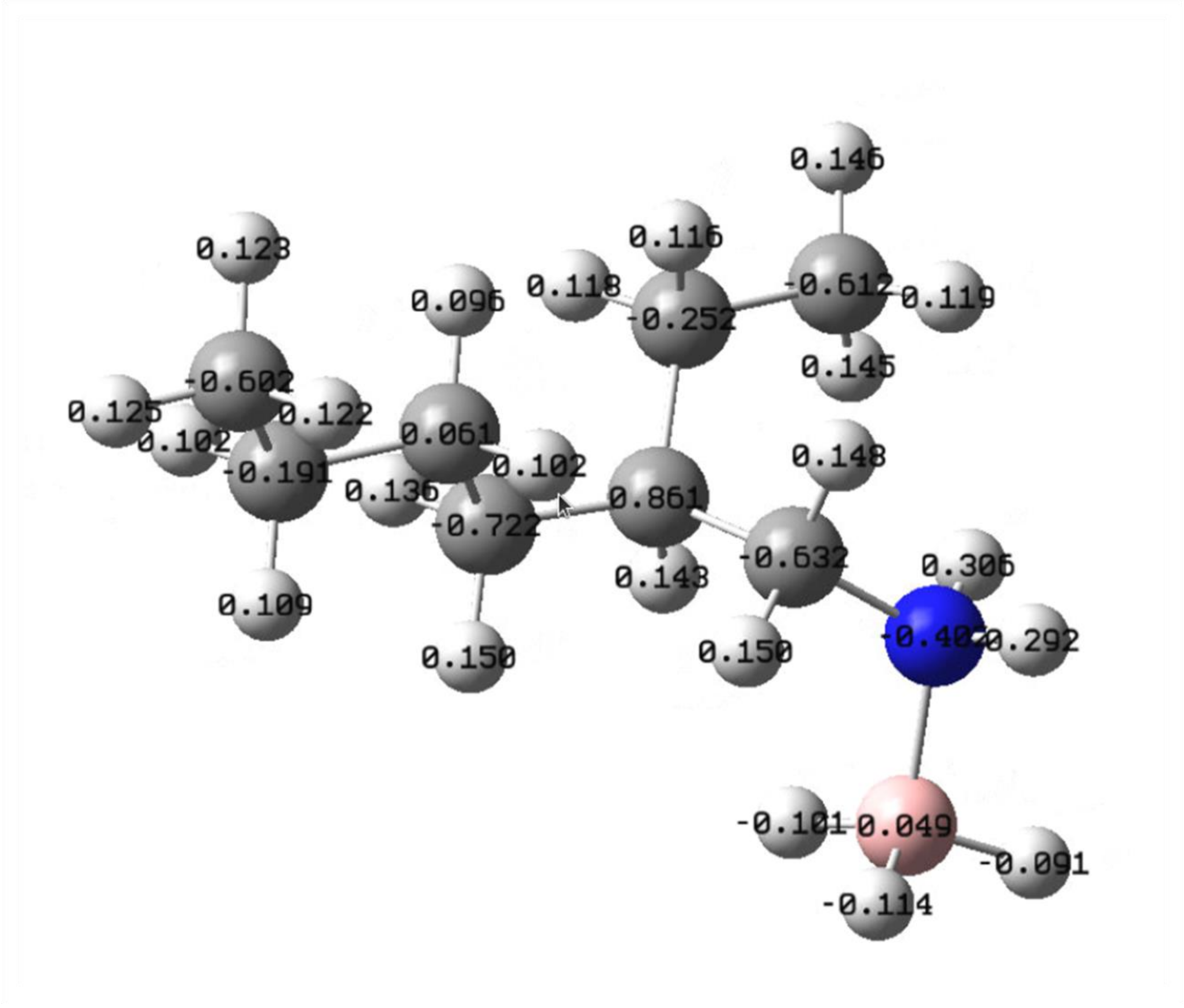

Figure S15. C2C6AB: partial charges extracted from Gaussian calculations following the Mulliken scheme. The H, B, N and C atoms are represented by white, pink, blue and dark grey spheres, respectively.

(C12)2AB: $[CH_3(CH_2)_{11}]_2NHBH_3$

Mulliken charges from DFT calculations

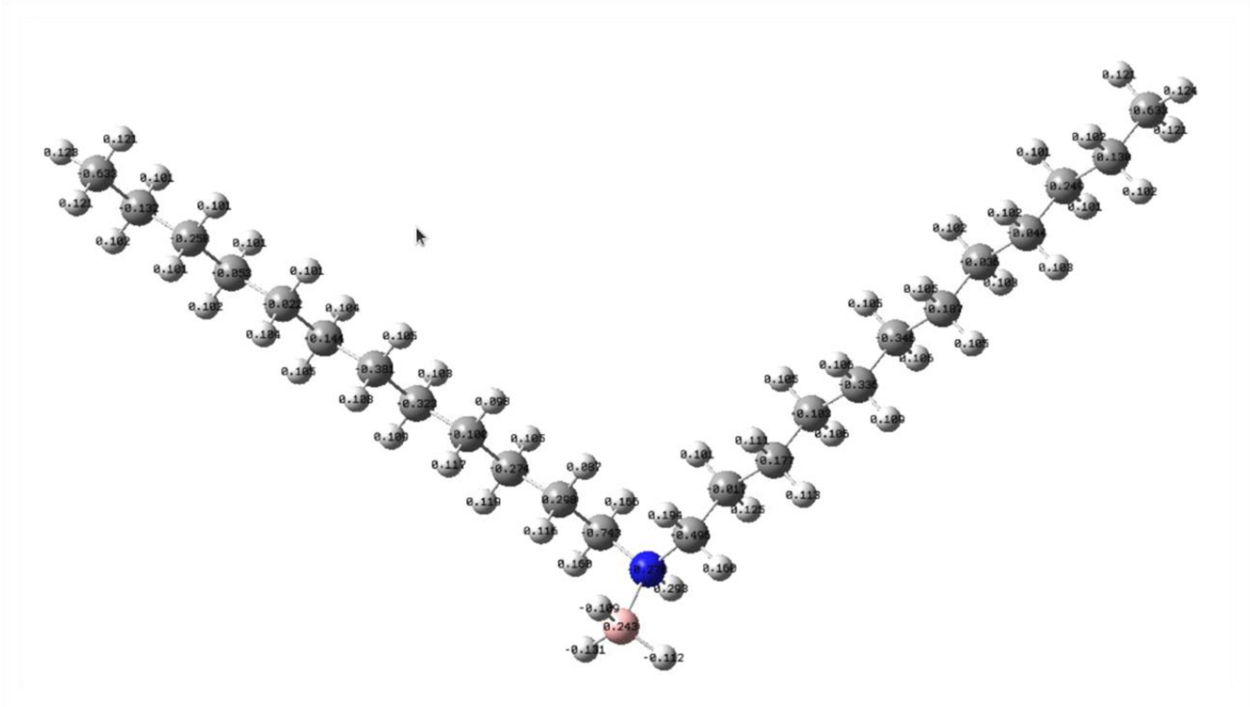

Figure S16. (C12)2AB: partial charges extracted from Gaussian calculations following the Mulliken scheme. The H, B, N and C atoms are represented by white, pink, blue and dark grey spheres, respectively

C3AB: $C_3H_5NH_2BH_3$

Electrostatic potentials from DFT calculations

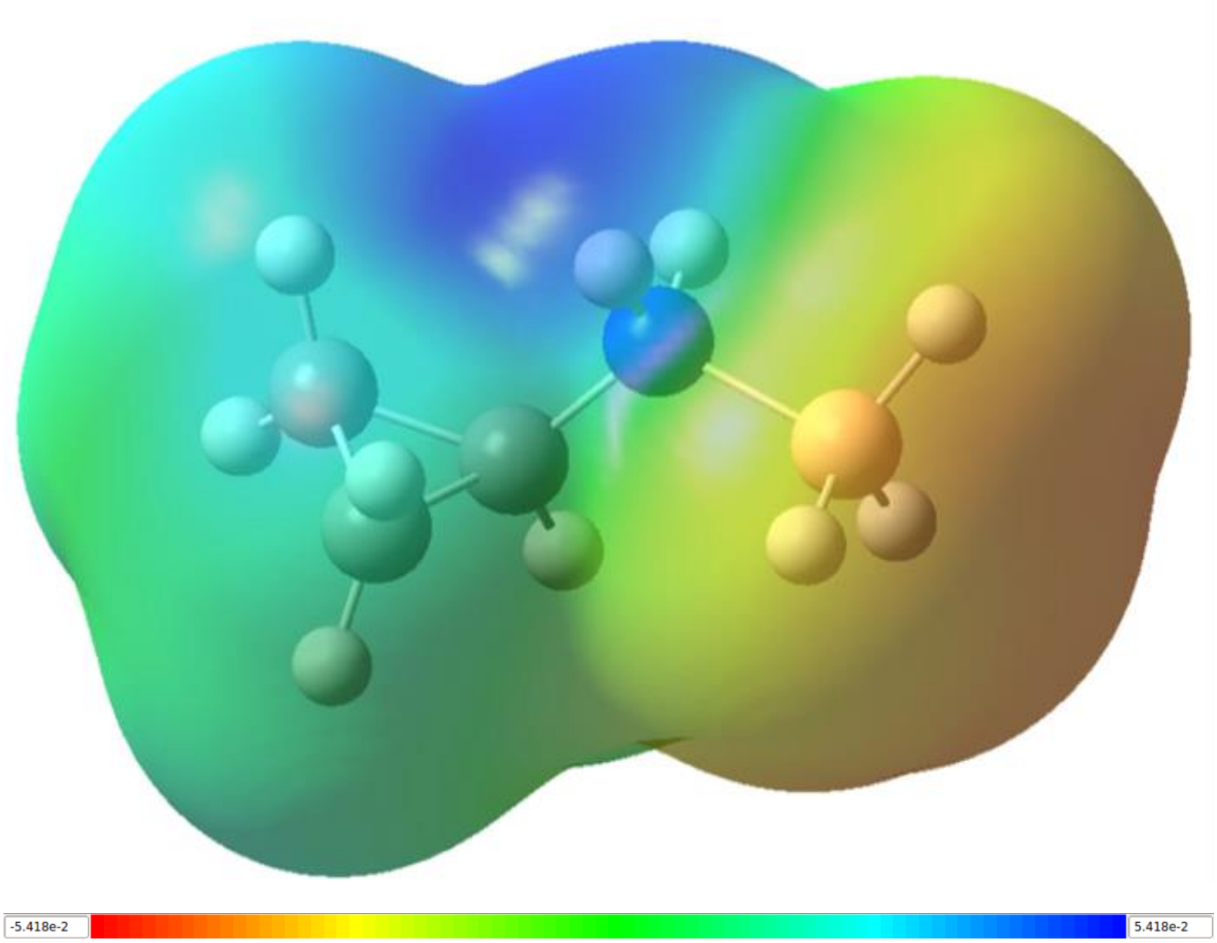

Figure S17. Electrostatic potentials of the C3AB molecule. The H, B, N and C atoms are represented by white, pink, blue and dark grey spheres, respectively.

C2C6AB: $CH_3(CH_2)_3CH(C_2H_5)CH_2NH_2BH_3$

Electrostatic potentials from DFT calculations

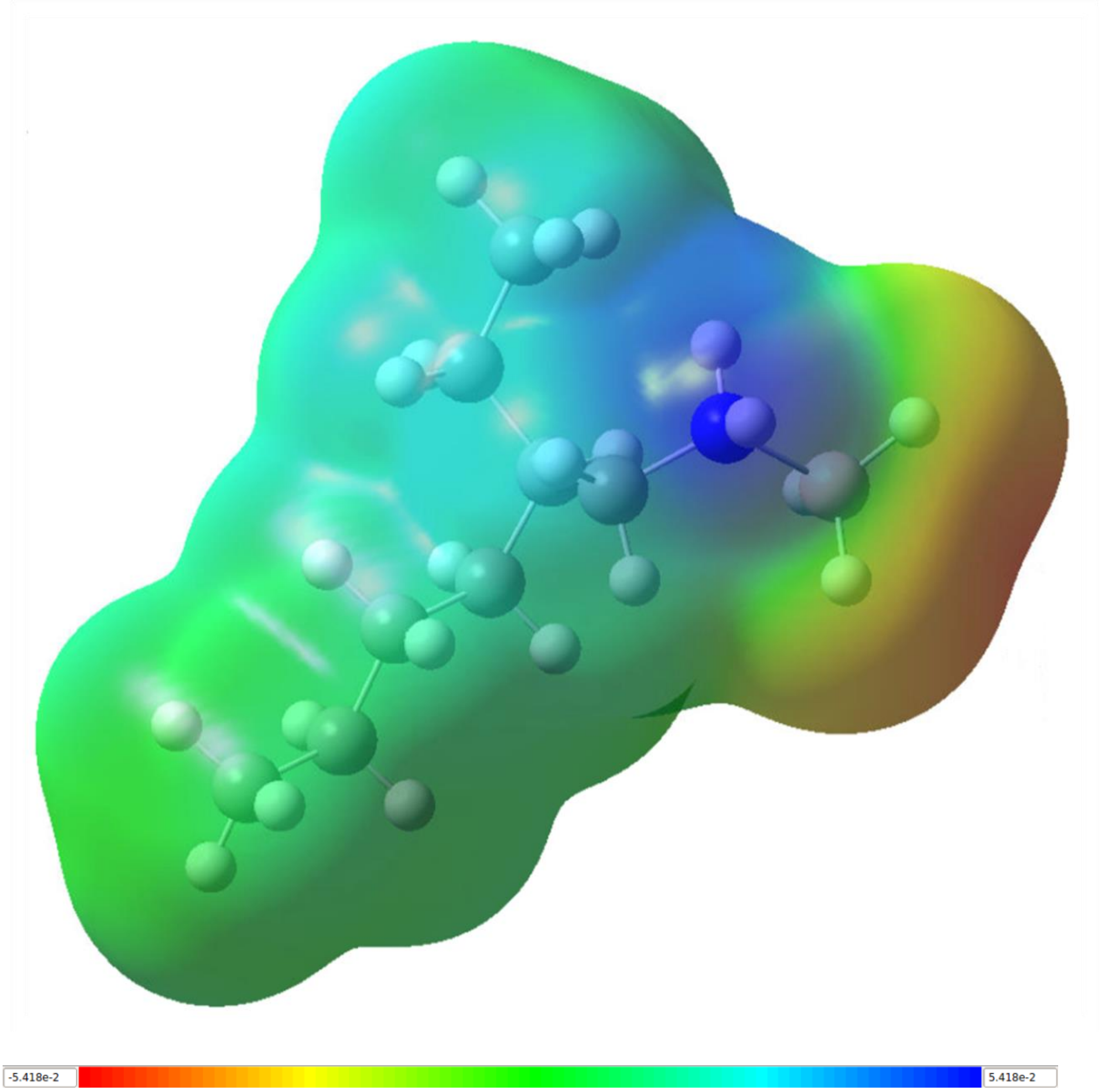

Figure S18. Electrostatic potentials of the C2C6AB molecule. The H, B, N and C atoms are represented by white, pink, blue and dark grey spheres, respectively.

(C12)2AB: $[CH_3(CH_2)_{11}]_2NHBH_3$

Electrostatic potentials from DFT calculations

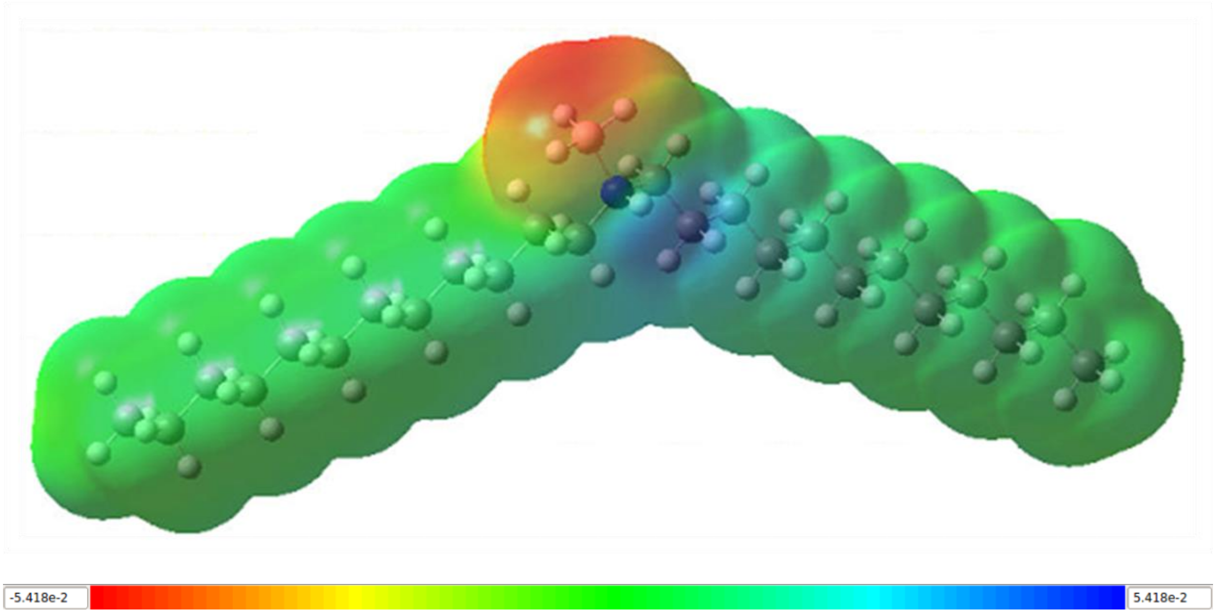

Figure S19. (Electrostatic potentials of the (C12)2AB molecule. The H, B, N and C atoms are represented by white, pink, blue and dark grey spheres, respectively.

C3AB: $C_3H_5NH_2BH_3$

TG and µGC analysis results

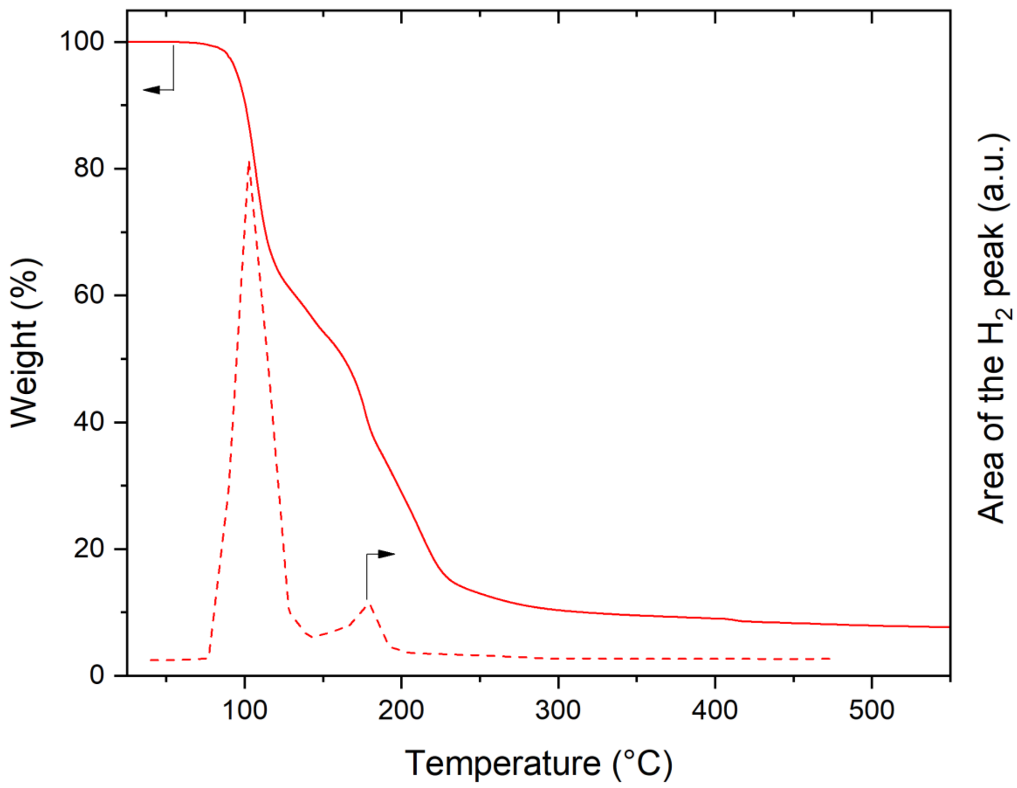

Figure S20. TG curve of C3AB superimposed with the evolution of H_2.as obtained by TGA/microGC-MS.

C3AB: $C_3H_5NH_2BH_3$

TG and DTG analysis results

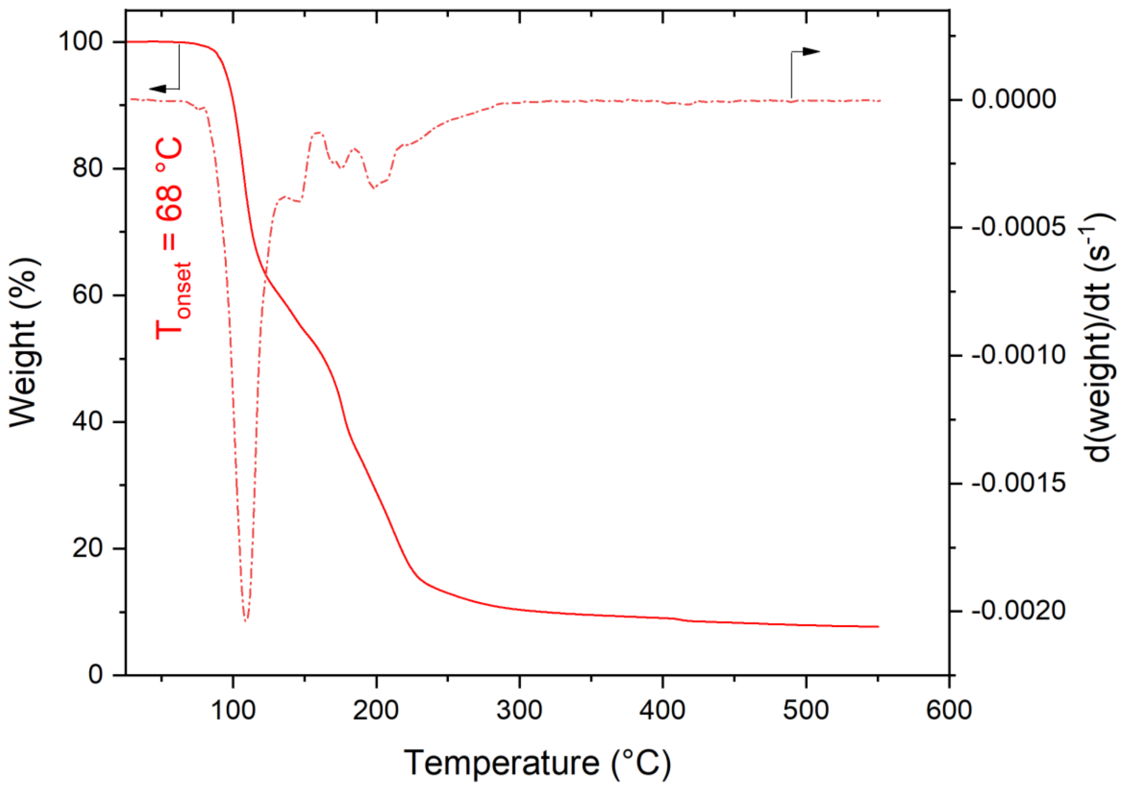

Figure S21. TG (left axis) and DTG (right axis) curves of C3AB.

C3AB: $C_3H_5NH_2BH_3$

µGC-MS analysis results

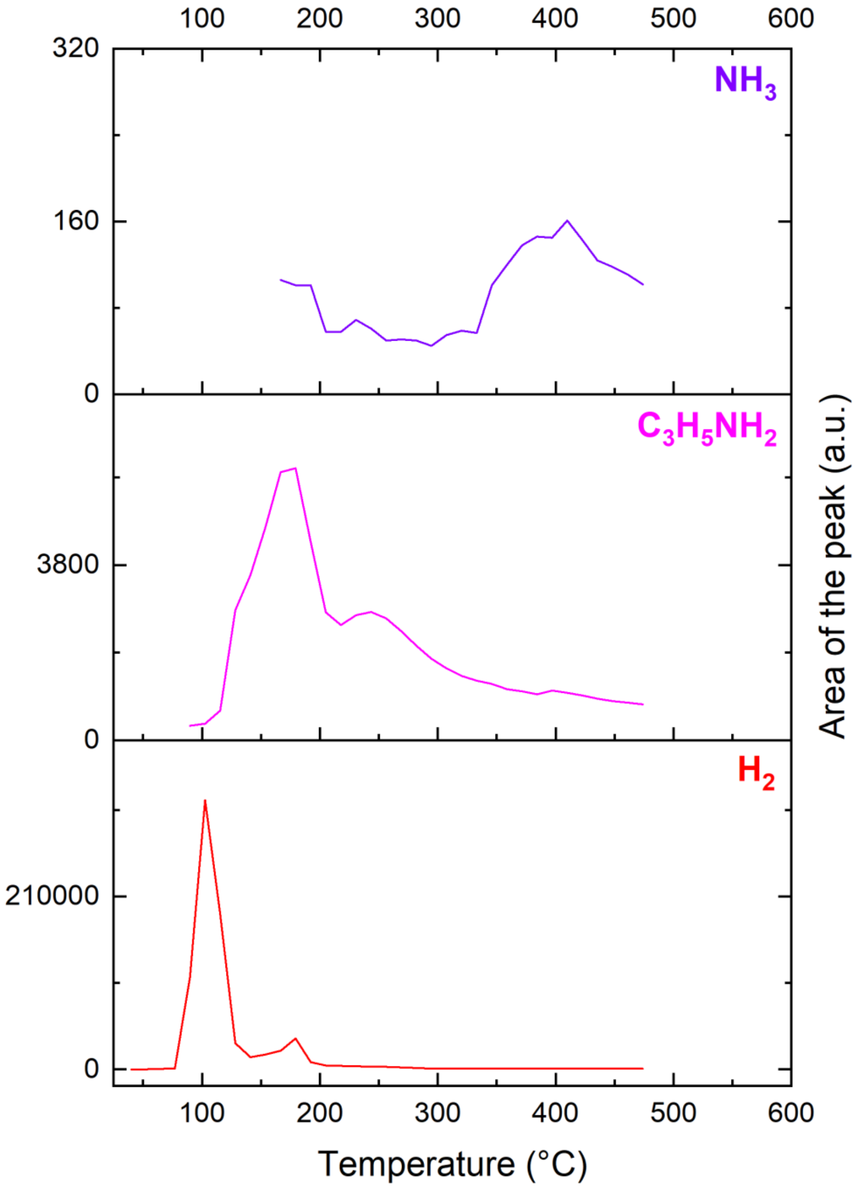

Figure S22. Thermal decomposition of C3AB: evolution of the peak area of the volatile products detected by TGA/microGC-MS coupling as a function of the sampling temperature. Hydrogen together with cyclopropylamine (from about 100 °C) and ammonia (from about 150 °C) were detected.

C2C6AB: $CH_3(CH_2)_3CH(C_2H_5)CH_2NH_2BH_3$

TG and µGC analysis results

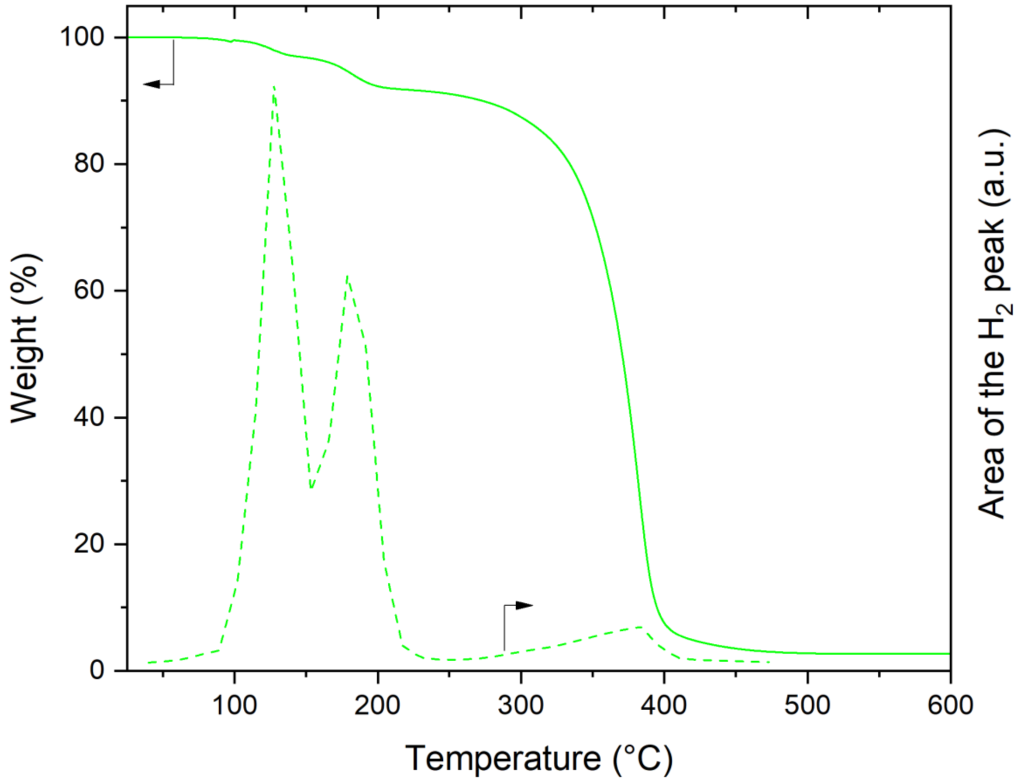

Figure S23. TG curve of C2C6AB, superimposed with the evolution of H_2 as obtained by TGA/microGC-MS.

C2C6AB: $CH_3(CH_2)_3CH(C_2H_5)CH_2NH_2BH_3$

TG and DTG analysis results

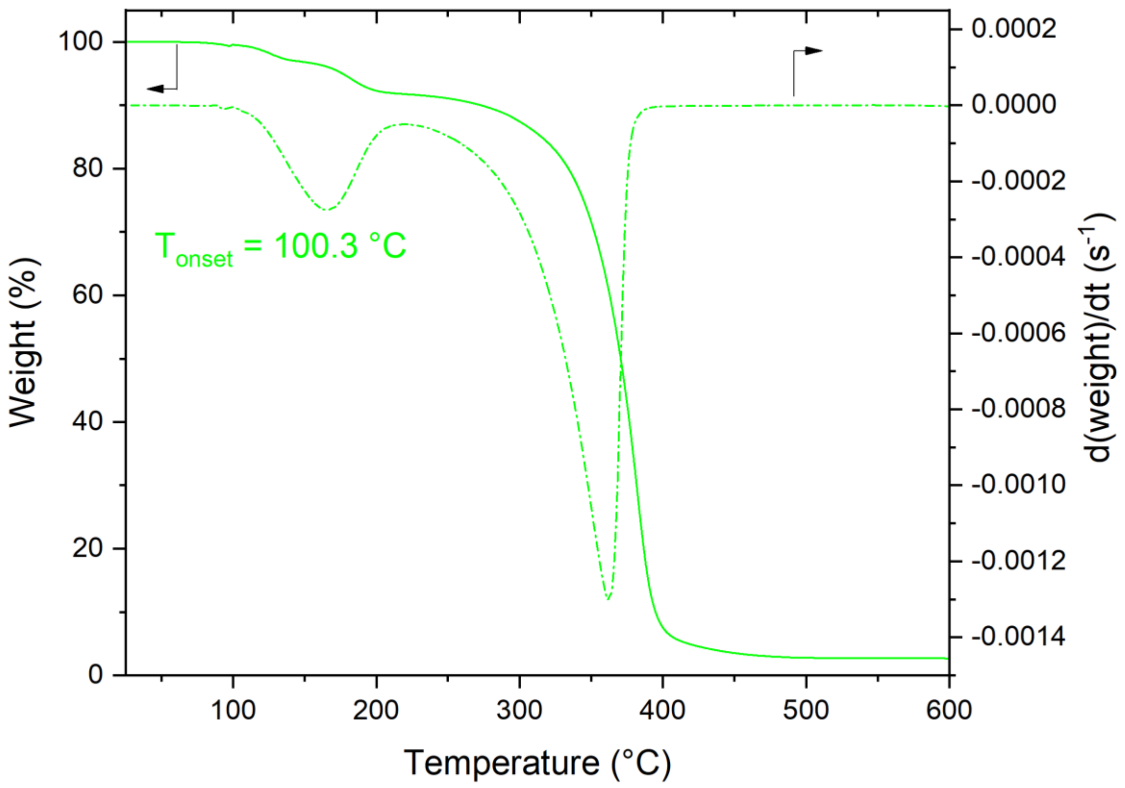

Figure S24. TG (left axis) and DTG (right axis) curves of C2C6AB.

(C12)2AB: $[CH_3(CH_2)_{11}]_2NHBH_3$

TG and µGC analysis results

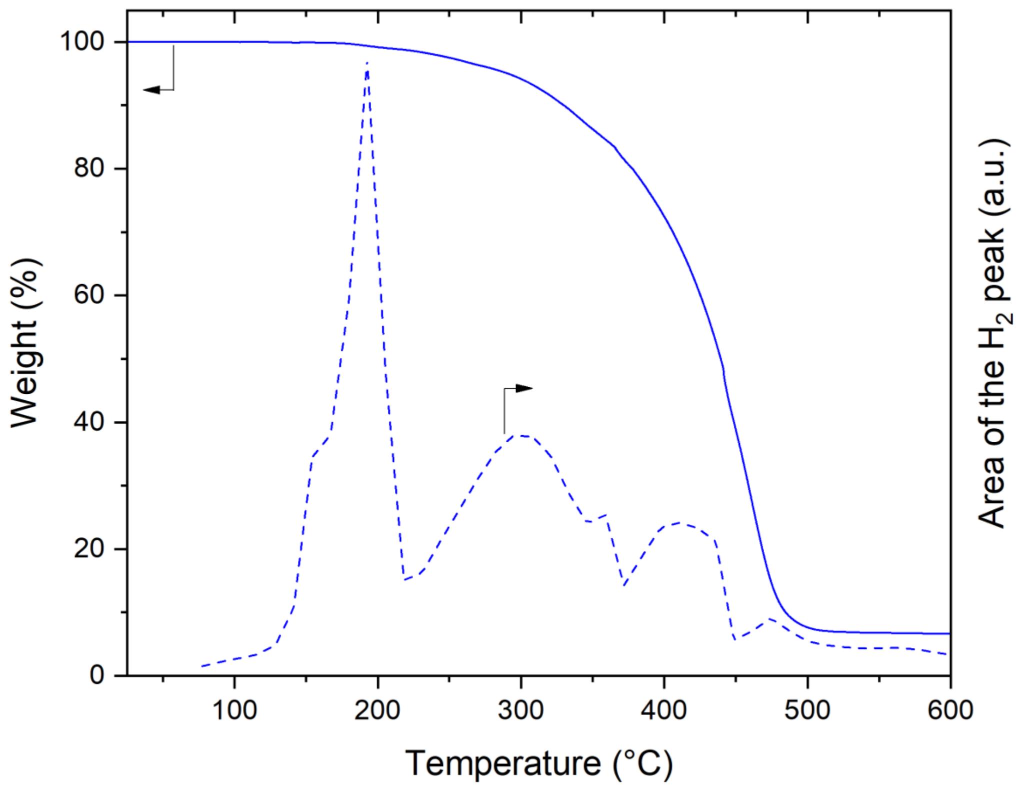

Figure S25. TG curve of (C12)2AB, superimposed with the evolution of H_2 as obtained by TGA/microGC-MS.The main weight loss between 200 and 600 °C are due to the release of hydrocarbon fragments of the alkyl chains.

(C12)2AB: $[CH_3(CH_2)_{11}]_2NHBH_3$

TG and DTG analysis results

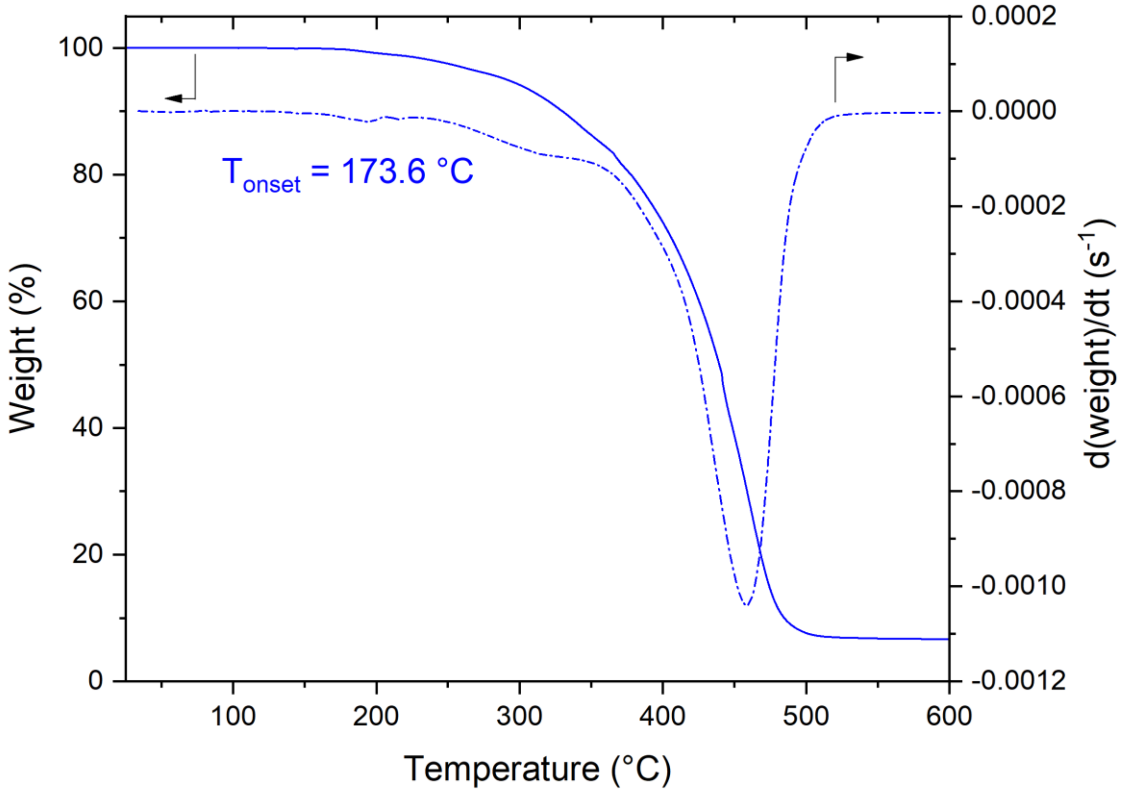

Figure S26. TG (left axis) and DTG (right axis) curves of (C12)2AB.

$(C12)2AB: [CH_3(CH_2)_{11}]_2NHBH_3$

MicroGC-MS and GC-MS results

Tableau S1. Volatile organic compounds obtained by coupling TGA with micro GC-MS and GC-MS during the decomposition of (C12)2AB.

Methane	Nonene
Butene	Nonane
Butane	Decene
2-Methyl-1-butene	Decane
Pentene	Undecene
Pentane	Undecane
Benzene	Dodecene
Hexadiene	Dodecane
Hexene	Tridecene
Hexane	Tridecane
Toluene	Tetradecene
Heptene	Tetradecane
Heptane	Dodecanitrile
Octene	Dodecylmethylamine
Octane	
